# Endothelial GTPCH (GTP Cyclohydrolase 1) and Tetrahydrobiopterin Regulate Gestational Blood Pressure, Uteroplacental Remodeling, and Fetal Growth

**DOI:** 10.1161/HYPERTENSIONAHA.120.17646

**Published:** 2021-10-25

**Authors:** Surawee Chuaiphichai, Grace Z. Yu, Cheryl M.J. Tan, Christopher Whiteman, Gillian Douglas, Yasmin Dickinson, Edward N. Drydale, Mahesh Appari, Wei Zhang, Mark J. Crabtree, Eileen McNeill, Ashley B. Hale, Adam J. Lewandowski, Nicholas J. Alp, Manu Vatish, Paul Leeson, Keith M. Channon

**Affiliations:** Division of Cardiovascular Medicine, British Heart Foundation Centre of Research Excellence, Radcliffe Department of Medicine, University of Oxford, United Kingdom (S.C., C.W., G.D., Y.D., E.N.D., M.A., M.J.C., E.M., A.B.H., N.J.A., K.M.C.).; Oxford Cardiovascular Clinical Research Facility, Division of Cardiovascular Medicine, Radcliffe Department of Medicine (G.Z.Y., C.M.J.T., A.J.L., P.L.), University of Oxford, United Kingdom.; Nuffield Department of Women’s and Reproductive Health (W.Z., M.V.), University of Oxford, United Kingdom.; National Institute for Health Research (NIHR) Oxford Biomedical Research Centre, Oxford University Hospitals National Health Service Foundation Trust, John Radcliffe Hospital, United Kingdom (M.V., K.M.C.).

**Keywords:** biopterin, blood pressure, endothelial cells, pregnancy, vascular remodeling

## Abstract

Abnormal uteroplacental remodeling leads to placental hypoperfusion, causing fetal growth restriction and pregnancy-related hypertension, which are associated with endothelial dysfunction and markers of reduced vascular NO bioavailability and oxidative stress. Tetrahydrobiopterin (BH4) is a redox cofactor for eNOS (endothelial NO synthase) with a required role in NO generation. Using mice models and human samples, we investigated the physiological requirement for endothelial cell BH4 in uteroplacental vascular adaptation and blood pressure regulation to pregnancy. In pregnant mice, selective maternal endothelial BH4 deficiency resulting from targeted deletion of *Gch1* caused progressive hypertension during pregnancy and fetal growth restriction. Maternal endothelial cell *Gch1* deletion caused defective functional and structural remodeling in uterine arteries and in spiral arteries, leading to placental insufficiency. Using primary endothelial cells isolated from either normal or hypertensive pregnancies, we found that hypertensive pregnancies are associated with reduced endothelial cell BH4 levels, impaired eNOS activity, and reduced endothelial cell proliferation, mediated by reduced GTPCH (GTP cyclohydrolase 1) protein. In rescue experiments, high blood pressure and fetal growth restriction in pregnant endothelial cell *Gch1* deficient mice was not rescued by oral BH4 supplementation, due to systemic oxidation of BH4 to dihydrobiopterin. However, the fully reduced folate, 5-methyltetrahydrofolate prevented BH4 oxidation, reduced blood pressure to normal levels, and normalized fetal growth. We identify a critical requirement for maternal endothelial cell BH4 biosynthesis in uteroplacental vascular remodeling in pregnancy. Restoration of endothelial cell BH4 with reduced folates identifies a novel therapeutic target for the prevention and treatment of pregnancy-related hypertension such as preeclampsia.

## Abstract 2

Supplemental Digital Content is available in the text.

**See Editorial, pp 1885–1887**

Vascular remodeling is a requirement for normal pregnancy, by providing adequate blood flow for placental perfusion that ensures fetal growth. Inadequate uteroplacental vascular remodeling leads to pregnancy-related hypertension and fetal growth restriction, which are major causes of adverse pregnancy outcomes, affecting ≈7% of all pregnancies worldwide.^1,2^ Furthermore, these factors have long-term effects on cardiovascular health in both mothers and offspring.^1–3^ Vascular adaptation in pregnancy requires remodeling of the uterine arteries and development of the placental vasculature sufficient to accommodate a 10-fold increase in uterine blood flow, without an increase in systemic blood pressure (BP).^4^ In placental insufficiency, uterine and placental vessels show increased medial vascular smooth muscle cell hypertrophy and reduced caliber, associated with increased plasma biomarkers related to endothelial cell dysfunction and abnormal angiogenesis, such as PIGF (placental growth factor)^5^ and soluble fms-like tyrosine kinase-1.^6^

In normal pregnancy increased uterine artery caliber is associated with enhanced activity of eNOS (endothelial NO synthase), and NO bioavailability,^7–9^ whereas endothelial dysfunction is a consistent finding in pregnancy-induced hypertension and uteroplacental insufficiency. NO-dependent flow-mediated vasodilatation is reduced in myometrial arteries isolated from hypertensive pregnancies (HTP).^10^ We have demonstrated that a feature of abnormal eNOS activity in cardiovascular disease states is loss of the required eNOS cofactor, tetrahydrobiopterin (BH4), that is synthesized in endothelial cells by the enzyme GTPCH (GTP cyclohydrolase 1), encoded by *GCH1*.^11,12^ However, the importance endothelial cell BH4 in uteroplacental remodeling and regulation of maternal BP during pregnancy is unknown.

Accordingly, we sought to investigate the notion that BH4 synthesis in the maternal endothelium has required roles in uteroplacental vascular remodeling and is hence a rational therapeutic target in pregnancy-associated hypertension, uteroplacental insufficiency, and fetal growth restriction.

## Methods

The authors declare that all supporting data are available within the article and its Supplemental Material. An extended Material and Methods section is available in the Supplemental Material.

### Generation of Endothelial Cell—Targeted *Gch1* Knockout Mice

We generated a novel mouse model of endothelial cell–specific BH4 deficiency, the *Gch1*^*fl/fl*^Tie2cre mouse. Exons 2 and 3 of *Gch1*, encoding for the active site of GTPCH, were flanked by locus of x-over, P1 sites in a targeting construct that was used to produce *Gch1*^*fl/fl*^ mice after homologous recombination in embryonic stem cells. These mice were crossed with Tie2cre transgenic mice to produce *Gch1*^*fl/fl*^Tie2cre mice where *Gch1* is deleted specifically in endothelial cells, generating an endothelial cell BH4-deficient mouse.^13^ Mice were housed in ventilated cages with a 12-hour light/dark cycle and controlled temperature (20 °C–22 °C) and fed normal chow and water ad libitum. Female *Gch1*^*fl/fl*^Tie2cre mice and their *Gch1*^*fl/fl*^ littermates (thereafter referred to as wild-type [WT]) were used for all experiments at 10 to 16 weeks. All studies were conducted in accordance with the UK Home Office Animals (Scientific Procedures) Act 1986 (HMSO, London, United Kingdom).

### Timed Mating

Pregnancy was achieved by mating either virgin female *Gch1*^*fl/fl*^Tie2cre or *Gch1*^*fl/fl*^ (WT) females (aged between 10 and 16 weeks old) with a *Gch1*^*fl/fl*^ male. To evaluate the gestation day, vaginal plugs were checked for the following morning, taken as the 0.5 day of gestation (E0.5). Body weights of plugged *Gch1*^*fl/fl*^Tie2cre and WT mice were determined throughout gestation (embryonic day [E]0, E2.5, E5.5, E7.5, E10.5, E12.5, E15.5, E16.5, E17.5, and E18.5). Urine samples from nonpregnant and pregnant (at E18.5) *Gch1*^*fl/fl*^Tie2cre and WT females were collected and stored at −80 °C for biochemistry analysis. Unless otherwise stated, all tissues were harvested and collected for experiments at either preconception (before timed mating) or E18.5 day of gestation (late gestation, one day before normal term delivery).

### BH4 and Biopterin Measurements

BH4 and oxidized biopterins (dihydrobiopterin [BH2] and biopterin) in plasma and uterine arteries (main branches from both sides) were determined by high-performance liquid chromatography (HPLC) followed by electrochemical and fluorescent detection, respectively, following established protocol.^11,14^

### BP Measurement by Implantable Telemetry

Nonpregnant female *Gch1*^*fl/fl*^Tie2cre and *Gch1*^*fl/fl*^ (WT) mice (8–10-week-old) underwent thoracic aortic implantation of telemeters (PAC10 radiotelemeters; DSI, Transoma Medical Inc.), as described previously.^15^

### Histology and Immunostaining

Placentas and uterine arteries from WT and *Gch1*^*fl/fl*^Tie2cre mice at E18.5 day of gestation were harvested following perfusion fixation at 100 mm Hg. Paraffin-embedded placentas and uterine arteries were stained with hematoxylin and eosin and immunohistochemistry for α-smooth muscle actin (Sigma), according to the manufacturer’s instructions.

### Clinical Cohort

Pregnant women under the care of the Oxford University Hospitals National Health Service Foundation Trust between 2011 and 2015 were invited to take part in clinical studies, as previously described.^3,16,17^ Mothers and infants were recruited from normotensive pregnancies, and pregnancy-induced hypertension, defined according to the International Society for the Study of Hypertension in Pregnancy guidelines.^18^ Blood samples were collected at the time of birth. All mothers gave written informed consent, as well as assent for involvement of their children, including permission to access maternal and offspring clinical records. Mothers below the age of 16 years were excluded from the study as were those with chronic cardiovascular conditions prenatally, including preexisting hypertension.^16^ Ethical approval was granted by South Central Berkshire Research Ethics Committee ref. 11/SC/0006, https://www.clinicaltrials.gov; Unique identifier: NCT01888770.^3,17^

### Isolation of Placental Extracellular Vesicles

Syncytiotrophoblast-derived extracellular vesicles were prepared using a modified dual-lobe placental perfusion system and differential centrifugation, as previously described.^19^ Briefly, placentae were perfused for 3 hours, and the maternal side perfusate was collected and immediately centrifuged (Beckman Coulter Avanti J-20XP centrifuge and Beckman Coulter JS-5.3 swing-out rotor) twice at 1500×*g* for 10 minutes at 4 °C to remove erythrocytes and large cellular debris. The supernatant was centrifuged at 150 000*g* for 3 hours to collect microvesicles and nanovesicles. Nanoparticle tracking analysis and flow cytometry were used as previously described to confirm the placental origin and size distribution of particles in the sample.^20^ After collection, the syncytiotrophoblast-derived extracellular vesicles were diluted in filtered PBS (4.9 mg protein/mL), and frozen (−80 °C) until further use in vascular experiments.

### Statistical Analysis

Data are presented as mean±SEM. Normality was tested using D’Agostino and Pearson omnibus normality test. Groups were compared using the Mann-Whitney *U* test for nonparametric data or an unpaired Student *t* test for parametric data. When comparing multiple groups, data were analyzed by ANOVA with Newman-Keuls post-test for parametric data or Kruskal-Wallis test with Dunns post-test for nonparametric data. When >2 independent variables were present a 2-way ANOVA with Tukey multiple comparisons test was used. When within-subject repeated measurements were present a repeated-measures ANOVA was used. A value of *P*<0.05 was considered statistically significant.

## Results

### Endothelial Cell–Specific *Gch1* Deletion Causes Pregnancy-Induced Hypertension and Fetal Growth Restriction in Female *Gch1*^*fl/fl*^Tie2cre Mice

To investigate the specific role of maternal endothelial cell BH4 in uteroplacental vascular remodeling and BP regulation during pregnancy, we investigated the response to pregnancy in female mice with endothelial cell–specific deletion of *Gch1*, encoding GTPCH. Endothelial cell–specific excision of the floxed allele was confirmed in uterine arteries and in the spiral arteries of the placental decidua, but not in other decidual cells, using fluorescence imaging of tissue sections from Tie2cre mice crossed with tdTomato reporter mice, at day 18.5 of pregnancy (Figure S1 in the Supplemental Material).

**Figure 1.**
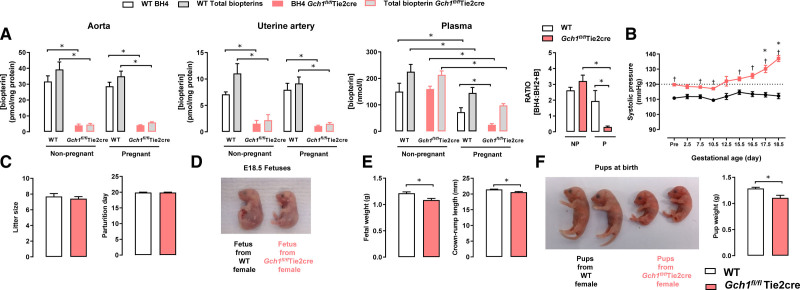
**Effects of endothelial cell–specific *Gch1* knockout in pregnancy.** Pregnancy was achieved by mating virgin wild-type (WT) and *Gch1*^*fl/fl*^Tie2cre females (aged between 10 and 16 wk old) with WT males. Tissues were harvested and collected for experiments at E18.5 d of gestation or from nonpregnant mice. **A**, Levels of biopterins in aortas, uterine arteries, and plasma from nonpregnant mice and pregnant (E18.5 d gestation) WT (*Gch1*^*fl/fl*^) and *Gch1*^*fl/fl*^Tie2cre mice were measured by high-performance liquid chromatography (HPLC). The tetrahydrobiopterin (BH4) and total biopterin levels were significantly decreased in *Gch1*^*fl/fl*^Tie2cre mice compared with WT mice in both nonpregnant and pregnant mice. In nonpregnant mice, there was no significant difference in plasma BH4 between WT and *Gch1*^*fl/fl*^Tie2cre mice. In pregnant mice, plasma BH4 levels were significantly reduced in both WT and *Gch1*^*fl/fl*^Tie2cre mice but with a greater extent in *Gch1*^*fl/fl*^Tie2cre mice such that the BH4/(dihydrobiopterin [BH2]+B) ratio was significantly decreased in *Gch1*^*fl/fl*^Tie2cre mice. The open (white) bars in each case are the levels of BH4, the gray-filled bars are the total biopterins (ie, BH4+BH2+B). *P<0.05; n=7–10 animals per group. **B**, Systolic blood pressure was measured by implantable telemeters in WT (*Gch1*^*fl/fl*^) and *Gch1*^*fl/fl*^Tie2cre mice before and during pregnancy. †*P*<0.05 comparing genotype; *P<0.05 comparing baseline blood pressure; n=5–7 animals per group. **C**, The number of fetuses per litter (litter size) or parturition day between WT and *Gch1*^*fl/fl*^Tie2cre mice. **D–E**, Fetuses from WT and *Gch1*^*fl/fl*^Tie2cre mothers were collected and weighed at E18.5 d of gestation (*P<0.05, n=72–85 pups from 10 to 13 litters per group). **F**, Offspring weights from WT and *Gch1*^*fl/fl*^Tie2cre mothers were determined at birth. Weights were averaged per litter of animals (*P<0.05, n=51–75 pups from 7 to 10 litters per group). B indicates biopterins.

In nonpregnant mice, BH4 and total biopterins levels in aortas and uterine arteries from *Gch1*^*fl/fl*^Tie2cre mice were significantly lower compared with that of WT mice (Figure 1A), whereas plasma levels of BH4 were not different between genotypes (Figure 1A), indicating that endothelial cell BH4 synthesis is not a major contributor to circulating biopterin levels in healthy nonpregnant female mice. In pregnant mice, BH4 and total biopterins levels in aortas and uterine arteries were comparable to nonpregnant mice from the same genotype (Figure 1A). However, plasma levels of BH4 and total biopterins were significantly reduced in pregnant mice both in WT and to a greater extent in *Gch1*^*fl/fl*^Tie2cre mice (Figure 1A). Furthermore, the BH4:BH2+B ratio in plasma was significantly reduced in pregnant *Gch1*^*fl/fl*^Tie2cre mice compared with nonpregnant *Gch1*^*fl/fl*^Tie2cre mice or pregnant WT mice (Figure 1A), indicating that endothelial cell–specific BH4 deficiency in pregnancy leads to further reduction in BH4 due to oxidation, forming BH2 and B. The reduction in plasma biopterins in pregnancy was not associated with any difference in liver biopterins, which is considered to be the principal source of circulating biopterins (Figure S2). In addition, plasma creatinine, urine creatinine, and renal histology were comparable between pregnant WT and *Gch1*^*fl/fl*^Tie2cre, indicating that endothelial cell *Gch1* and BH4 deletion do not exert effects through changes in renal function (Figures S3 and S4).

We next determined the requirement for maternal endothelial cell BH4 biosynthesis in BP regulation during pregnancy. We evaluated BP changes using both implantable telemeters (implanted before pregnancy) and tail-cuff plethysmography. As previously reported,^13^ systolic BP and mean BP were slightly higher (≈7 mm Hg) in female nonpregnant *Gch1*^*fl/fl*^Tie2cre mice, compared with nonpregnant WT littermates (Figure 1B and Figure S5). However, by day E17.5 of gestation, systolic BP and mean BP were significantly increased above basal levels and further elevated at E18.5 day of gestation in pregnant *Gch1*^*fl/fl*^Tie2cre mice compared to those of nonpregnant *Gch1*^*fl/fl*^Tie2cre mice (Figure 1B and Figure S5). By the end of pregnancy, diastolic BP in *Gch1*^*fl/fl*^Tie2cre mice was also significantly increased (Figure S5).

To address the potential effect of the small increase in baseline BP in *Gch1*^*fl/fl*^Tie2cre mice, we first analyzed additional cohorts of *Gch1*^*fl/fl*^Tie2cre and WT mice that were matched for baseline BP. In *Gch1*^*fl/fl*^Tie2cre mice with prepregnancy BPs that were identical to a paired cohort of WT mice, the increase in BP during pregnancy was significantly increased (23±5 mm Hg), whereas the cohort of WT demonstrated no increase in BP during pregnancy (Figure S6).

To determine the importance of maternal endothelial cell BH4 on placental and fetal development, we determined the weight and size of placentas and offspring born to *Gch1*^*fl/fl*^Tie2cre and WT mice. There were no significant differences in the number of pups per litter between *Gch1*^*fl/fl*^Tie2cre and WT mice at E18.5 day of gestation or at birth, nor was the onset of parturition different between *Gch1*^*fl/fl*^Tie2cre and WT mice (Figure 1D). In contrast, we found that fetuses at day E18.5 were significantly smaller (≈10%) from pregnant *Gch1*^*fl/fl*^Tie2cre females compared with WT females (Figure 1D, 1E, and 1F). There was a corresponding reduction in crown-to-rump length of fetuses from pregnant *Gch1*^*fl/fl*^Tie2cre mice (Figure 1E). In keeping with the reduction in fetal size at E18.5, mean body weight of offspring born at term from *Gch1*^*fl/fl*^Tie2cre females was significantly lower than that of offspring from WT mice (Figure 1F).

**Figure 2.**
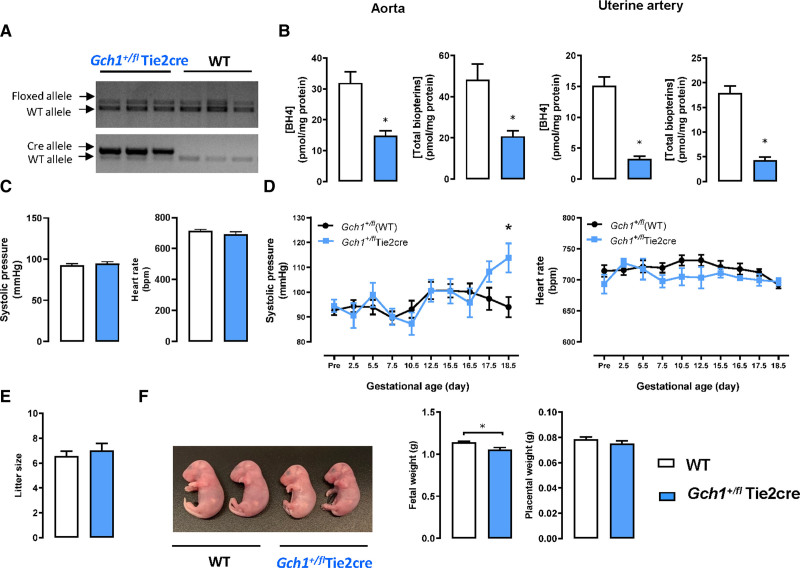
**Biopterin levels, blood pressure, and fetal growth in pregnant mice with heterozygous deletion of *Gch1* in endothelial cells (ie, *Gch1*^*fl/+*^ Tie2cre mice).** Mice with heterozygous deletion of *Gch1* in endothelial cells (ie, *Gch1*^*fl/+*^ Tie2cre mice) were generated by crossing *Gch1*^*fl/fl*^ Tie2cre mice with wild-type (WT; ie, *Gch1*^+/+^) mice. Female *Gch1*^*fl/+*^ Tie2cre mice were mated with WT male mice. **A**, Genomic polymerase chain reaction shows the presence of the *Gch1* floxed and WT alleles in both *Gch1*^*fl/+*^ (WT) and *Gch1*^*fl/+*^ Tie2cre mice. **B**, Levels of tetrahydrobiopterin (BH4), and total biopterins were measured by high-performance liquid chromatography (HPLC) in tissue homogenates obtained from WT and *Gch1*^*fl/+*^ Tie2cre mice, at the end of pregnancy. Total biopterins include BH4+dihydrobiopterin (BH2)+B. Shown are biopterin levels in aorta, pregnant uterine artery. *P<0.05 vs WT. **C**, Blood pressure and heart rate at baseline (prepregnancy; n=6–8 animals per group). **D**, Blood pressure and heart rate before and during pregnancy (n=6–8 animals per group). **E**, Litter size (number of embryos) was not different between WT and *Gch1*^*fl/+*^ Tie2cre mice (n=6–8 animals per group). **F**, Fetuses at gestation day 18.5 were significantly smaller from pregnant *Gch1*^*fl/+*^ Tie2cre compared with WT females. *P<0.05 vs WT; n=6–8 animals per group. B indicates biopterins.

To test the effects of more moderate endothelial cell BH4 deficiency in pregnancy, we next generated cohorts of mice with endothelial cell–specific deletion of only one *Gch1* allele, that is, *Gch1*^*fl/+*^ Tie2cre mice to investigate whether loss of a single *Gch1* would be sufficient to cause endothelial cell BH4 deficiency, and if so, the effect on BP. We found that BH4 levels in aorta and lung of *Gch1*^*fl/+*^ Tie2cre mice were reduced by ≈50% compared with WT mice, less than the ≈80% reduction in BH4 observed in *Gch1*^*fl/fl*^Tie2cre mice (Figure 2A and 2B, and Figure S7). Levels of BH4 in liver were unchanged in *Gch1*^*fl/+*^ Tie2cre mice (Figure S7). In pregnant *Gch1*^*fl/+*^ Tie2cre mice, BH4 levels were reduced by ≈80% in uterine arteries (Figure 2B).

BP measurements in *Gch1*^*fl/+*^ Tie2cre mice revealed no difference in baseline (prepregnancy) BP (Figure 2C), but a significant increase in BP during late pregnancy was still observed in *Gch1*^*fl/+*^ Tie2cre mice, despite the normal baseline BP and modest level of endothelial cell BH4 deficiency (Figure 2D). We further evaluated the effect of heterozygous loss of *Gch1* in maternal endothelial cells on fetal growth, in pregnant *Gch1*^*fl/+*^ Tie2cre mice, crossed with WT male mice to generate litters with only WT and *Gch1*^*fl/+*^ Tie2cre fetuses (ie, no fetuses with homozygous deletion of endothelial cell *Gch1*). Offspring from pregnant *Gch1*^*fl/+*^ Tie2cre mice were significantly smaller than those from WT females (Figure 2E and 2F). These data indicate that the hypertensive response to pregnancy induced by maternal endothelial cell BH4 deficiency is dose-dependent and is not dependent on the small increase in baseline BP in *Gch1*^*fl/fl*^ Tie2cre mice.

To distinguish the role of maternal endothelial cell BH4 biosynthesis from that of fetal BH4, WT females were mated with *Gch1*^*fl/fl*^Tie2cre males, and *Gch1*^*fl/fl*^Tie2cre females were mated with WT males to generate pregnant female mice with matched litters of equal proportions of WT and *Gch1*^*fl/fl*^Tie2cre offspring (for breeding strategy, see Figure S8). Only *Gch1*^*fl/fl*^Tie2cre females developed progressive hypertension during pregnancy, whereas WT females mice mated with *Gch1*^*fl/fl*^Tie2cre males had normal BP during pregnancy, despite bearing identical litters of WT and *Gch1*^*fl/fl*^Tie2cre offspring (Figure S8**).** Similarly, the reduction in fetal size was dependent solely on maternal *Gch1*^*fl/fl*^Tie2cre genotype. There was no difference in the reduction in fetal weight between male and female fetuses from *Gch1*^*fl/fl*^Tie2cre mice (Figure S9). These findings show that lack of maternal, but not fetal, endothelial cell BH4 causes pregnancy-induced hypertension and is responsible for reduced fetal growth.

### Endothelial Cell BH4 Is Required for Structural Uteroplacental Remodeling in Pregnancy

**Figure 3.**
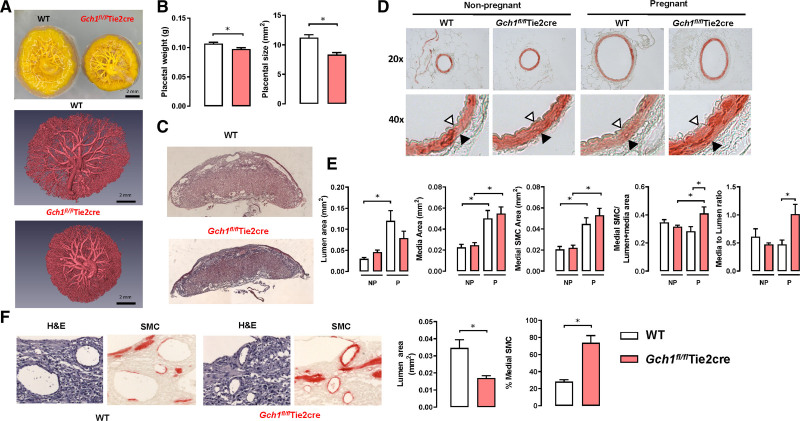
**Effect of endothelial cell tetrahydrobiopterin (BH4) deficiency on placental size and vascular remodeling in uterine arteries and spiral arteries in pregnancy.** Vascular remodeling was analyzed in embedded sections of uterine arteries (perfusion fixed at 100 mm Hg) and placentas from nonpregnant and pregnant wild-type (WT) and *Gch1*^*fl/fl*^Tie2cre mice. **A**, Representative images of placental casts of the umbilical arterial and venous circulation from WT (**left**) and *Gch1*^*fl/fl*^Tie2cre mice (**right**) at E18.5 d of gestation (**top**). Representative micro–computed tomography images (superior view) of placental casts of umbilical arterial and venous circulation from WT and *Gch1*^*fl/fl*^Tie2cre mice (**right**) at E18.5 d of gestation (**bottom**). **B**, Placentas from wild-type and *Gch1*^*fl/fl*^Tie2cre mothers were collected and weighed at E18.5 d of gestation (*P<0.05, n=72–85 pups from 10 to 13 litters per group). **C**, Hematoxylin and eosin (H&E) staining of a representative mouse placental sections from WT and *Gch1*^*fl/fl*^Tie2cre dams. Quantification of placental area was performed by using Image Pro Plus image analysis software. *P<0.05, n=6–8 per group. **D**, Representative images are shown α-SMA (α-smooth muscle actin) staining of uterine arteries. Opened arrow indicates internal elastic lamina and closed arrow indicates external elastic lamina. **E**, Vascular remodeling and medial hypertrophy in uterine artery sections from nonpregnant (NP) and pregnant (P) WT and *Gch1*^*fl/fl*^ Tie2cre mice. Vascular remodeling was evaluated by quantification of lumen area, media area, vascular smooth muscle (VSM) area (by α-SMA immunostaining), VSM to lumen+media ratio, and media to lumen ratio. Quantification was performed by using Image Pro Plus software (*P<0.05; n=5–6 animals per group). **F**, H&E and α-SMA staining of representative spiral arteries in the decidua of mouse placenta from WT and *Gch1*^*fl/fl*^Tie2cre mice. Quantification of lumen area and percentage medial smooth muscle cells (SMCs) of spiral arteries was quantified by α-SMA immunostaining and Image Pro Plus software. *P<0.05; n=5 animals per group.

To determine the effects of endothelial cell BH4 deficiency on structural vascular remodeling in pregnancy, we compared uterine arteries and placental spiral arteries from WT and *Gch1*^*fl/fl*^Tie2cre mice. Placental weights and placental size from *Gch1*^*fl/fl*^Tie2cre pregnancies at gestational day E18.5 day were significantly reduced compared with those from WT pregnant females (Figure 3A through 3C). In uterine arteries, pregnancy caused the expected marked increase in medial area, luminal diameter, and luminal area in uterine arteries of both WT and *Gch1*^*fl/fl*^Tie2cre mice compared to nonpregnant mice (Figure 3D and 3E), reflecting the increase in uterine blood flow during pregnancy. However, these changes were significantly impaired in uterine arteries of *Gch1*^*fl/fl*^Tie2cre mice (Figure 3D and 3E), with a smaller increase in luminal area, increased medial area and the area of vascular smooth muscle actin immunostaining, and a significant increase in media to lumen ratio. Furthermore, we found that decidual spiral arteries in placentas from *Gch1*^*fl/fl*^Tie2cre mice failed to undergo the remodeling observed in WT mice, as indicated by reduced luminal area, and by increased muscularization, revealed by immunohistochemistry for vascular smooth muscle cell alpha-actin (Figure 3F). These findings demonstrate that selective endothelial cell BH4 deficiency causes impaired uteroplacental vascular remodeling in the maternal physiological response to pregnancy, in both uterine conduit arteries and placental resistance vessels.

### Pregnancy-Induced Hypertension Is Associated With Reduced Endothelial Cell GTPCH and BH4 Levels, Impaired NOS Activity, and Impaired Endothelial Tube Formation

**Figure 4.**
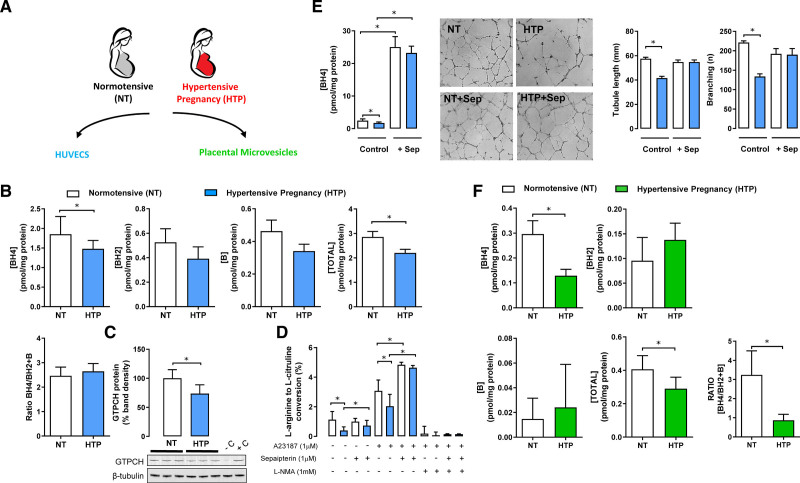
**GTPCH (GTP cyclohydrolase 1) protein, tetrahydrobiopterin (BH4) levels, NOS (NO synthase) activity, and in vitro endothelial tube formation in endothelial cells from normotensive and hypertensive pregnancies.** **A**, Schematic diagram showing human umbilical vein endothelial cells (HUVECs) and placental extracellular vesicles were isolated from umbilical cords of placentas and from perfused placentas, respectively, from either normotensive (NT) or hypertensive pregnancies (HTP). **B**, High-performance liquid chromatography (HPLC) analysis of biopterins in HUVECs from NT and HTP. Biopterins are expressed per mg of cellular protein (*P<0.05; n=12–14 per group). **C**, Representative immunoblots showing GTPCH protein in HUVECs from NP and HTP pregnancies, with band density quantified relative to β-tubulin loading control (*P<0.05; n=9 per group). The identity of bands obtained was confirmed using control lysates from HUVECs treated with siRNA *GCH1* (as negative control) or with nonspecific siRNA (as positive control). **D**, eNOS (endothelial NOS) activity was measured by conversion of ^14^C arginine in cell culture, followed by radiochemical HPLC quantification of ^14^C citrulline production. eNOS activity was greatly reduced in HUVECs from HTP compared with NT under basal conditions and when stimulated with calcium ionophore (A23187). Treatment with sepiapterin (Sep; 1 μM, converted to BH4 by the cellular pterin salvage pathway) significantly increased eNOS activity in both NT and HTP HUVECs, such that eNOS activity was no longer different between the groups. The nonselective NOS inhibitor *N*G-methyl-L-arginine (L-NMA), abolished eNOS activity in all groups (*P<0.05; n=6–8 per group). **E**, The levels of BH4 in HUVECs from NT and HTP treated with or without 1 μM Sep. Representative photomicrographs of HUVECs from NT and HTP plated on growth factor-reduced matrigel in the presence or absence of Sep (1 μM). **E**, Quantification of endothelial branches points and total tube length was performed using Angiosys software and expressed in micrometers per field. HUVECs from HTP showed lesser endothelial cell growth and tubule formation than HUVECs from NT but were rescued by supplementation with Sep (*P<0.05; n=6 per group). **F**, Extracellular vesicles were isolated by dual-lobe placental perfusion and ultracentrifugation from placentas from women with NT or from women with HTP. The levels of BH4, BH2, and total biopterins and ratio of BH4 relative to oxidized biopterin species (BH4:BH2+B) were measured by HPLC. BH4, total biopterins, and BH4/total biopterin ratio were significantly reduced in extracellular vesicles isolated from women with hypertensive pregnancies compared to women with normotensive pregnancies (*P<0.05; n=6 patients per group). B indicates biopterins.

Given the findings showing a requirement for endothelial cell *Gch1* and BH4 in the responses to pregnancy in mice, we next sought to investigate whether endothelial *GCH1* and BH4 are altered in pregnancy-induced hypertension in humans. We measured BH4 levels in primary human umbilical vein endothelial cells (HUVECs) and placental extracellular vesicles isolated from perfused placentas obtained from women who had pregnancies complicated by hypertension (HTP), in comparison with mothers with normotensive pregnancies (Figure 4A). The clinical characteristics of the study participants are shown in Table S1. Mothers with hypertension had higher BP, and elevated levels of soluble endoglin at 5 days postpartum (Figure S10).

We found that the levels of BH4, GTPCH protein, and eNOS activity were significantly decreased in HUVECs from HTP compared with endothelial cells from normotensive pregnancies (Figure 4B through 4D). Furthermore, endothelial cell tube formation, a marker of endothelial cell growth, was reduced in HUVECs from HTP (Figure 4E). To test the dependence of these endothelial cell abnormalities on BH4, incubation with the BH4 precursor, sepiapterin, normalized both BH4 levels and NOS activity in HUVECs from HTP, such that BH4 levels and NOS activity were no longer different between the groups (Figure 4D and 4E). Furthermore, sepiapterin restored tube formation in HUVECs from HTP (Figure 4E). In contrast to the reduced levels of BH4 in endothelial cells from HTP, circulating plasma BH4 levels and total biopterins were significantly higher in mothers with HTP compared with controls (Figure S11). Furthermore, plasma BH4 levels in both normal and HTP were significantly decreased in late pregnancy compared with baseline (Figure S11).

**Figure 5.**
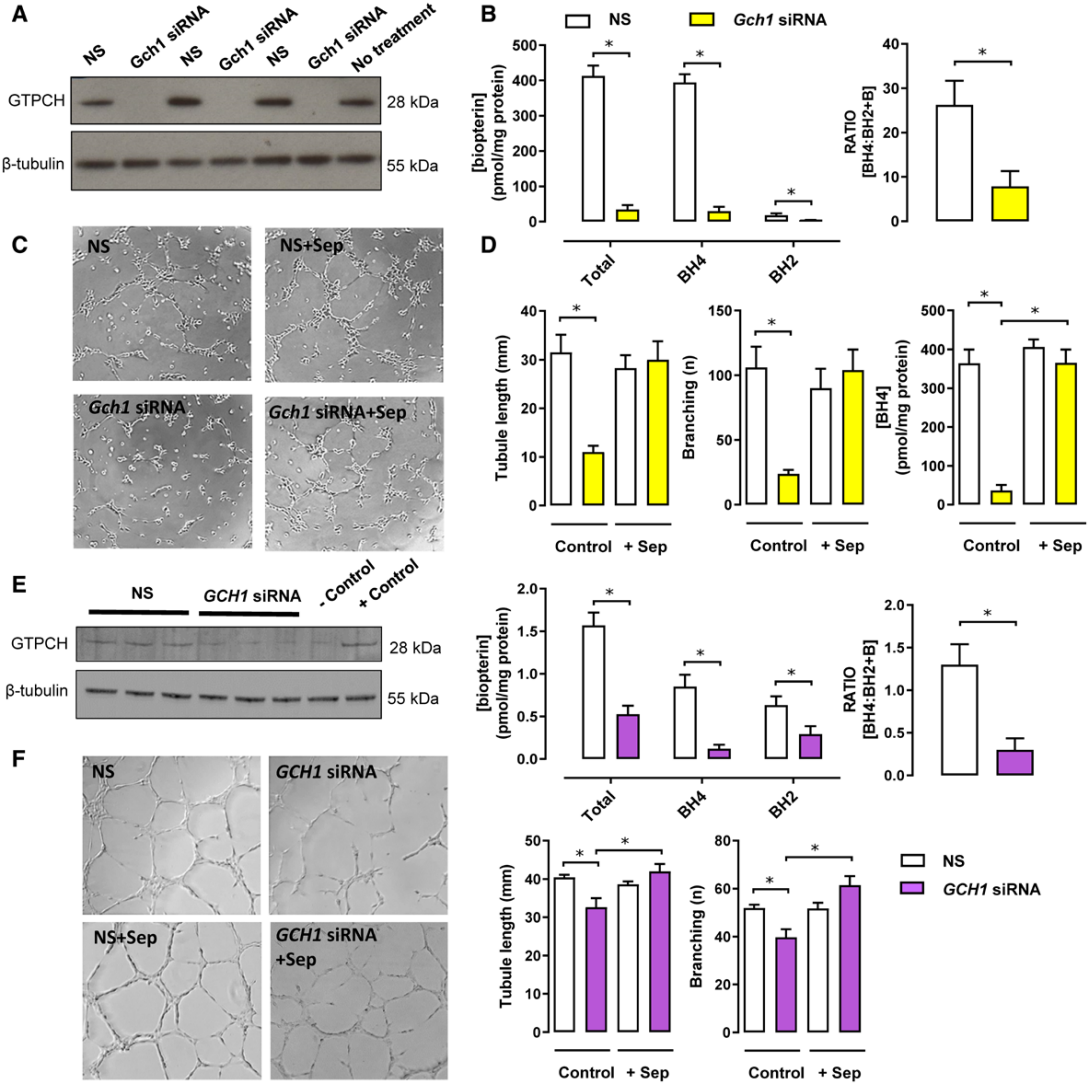
**Effect of *Gch1* knockdown on in vitro endothelial tube formation in endothelial cells.** sEnd.1 murine endothelial cells (**A–D**) and primary human uterine microvascular endothelial cells (HutMECS; **E** and **F**) were transfected with a siRNA pool targeted to *Gch1* or a nontargeting (nonspecific; NS) scrambled control siRNA. The cells were then harvested and analyzed for GTPCH (GTP cyclohydrolase 1) protein expression by Western blotting, or biopterin levels using high-performance liquid chromatography (HPLC) with electrochemical and fluorescent detection. **A**, Representative Western blot for GTPCH protein in sEND.1 mouse endothelial cell line treated with NS *Gch1* siRNA or specific *Gch1* siRNA (*Gch1* siRNA). In sEND.1 treated with specific *Gch1* siRNA, GTPCH protein was not detectable by Western blotting, whereas GTPCH was readily detected in sEND.1 treated with NS *Gch1* siRNA. The blot is representative of four separate experiments. **B**, Intracellular tetrahydrobiopterin (BH4), dihydrobiopterin (BH2), total biopterins, and ratio of BH4 relative to oxidized biopterin species (BH4:BH2+B), measured by HPLC, were significantly reduced in *Gch1*-specific siRNA cells compared with NS siRNA cells (*P<0.05; n=4 per group). **C**, Representative photomicrographs of sEND.1 mouse endothelial cell treated with NS *Gch1* siRNA or specific *Gch1* siRNA plated on growth factor-reduced matrigel in the presence or absence of sepiapterin (Sep; 1 μM). Quantification of endothelial tube length and branching was performed by using Angiosys software and expressed in micrometers per field. **D**, In vitro endothelial tube formation and branching. Reduction of *Gch1* expression in sEND.1 treated with specific *Gch1* siRNA caused a marked decrease in endothelial cell growth and tubule formation compared to sEND.1 treated with NS *Gch1* siRNA, which can be rescued by supplementation with Sep (*P<0.05; n=6 per group). The levels of BH4 in sEND.1 cells treated with NS *Gch1* siRNA or specific *Gch1* siRNA in the presence or absence of Sep (1 μM; *P<0.05; n=6 per group). **E**, Western blot analysis shows that cellular GTPCH protein was greatly reduced in HutMECs following exposure to *GCH1*-specific siRNA, compared to NS siRNA. The identity of bands obtained was confirmed using control lysates from human umbilical vein endothelial cells (HUVECs) treated with siRNA *GCH1* (as negative control) or with NS siRNA (as positive control). *GCH1* specific siRNA significantly reduced the detectable levels of the cellular biopterins and the ratio of BH4 to BH2+B (*P<0.05; n=4 per group). **F**, Representative photomicrographs of HutMECs treated with NS *GCH1* siRNA or specific *GCH1* siRNA plated on growth factor-reduced matrigel in the presence or absence of Sep (1 μM). Quantification of endothelial tube length and branching was performed by using Angiosys software and expressed in micrometers per field. In vitro endothelial tube formation and branching. Reduction of *GCH1* expression in human uterine endothelial cells caused a significant decrease in endothelial cell growth and tubule formation, which can be rescued by supplementation with Sep (1 μM; *P<0.05; n=4 per group). B indicates biopterins.

To further investigate the relevance of BH4 in the human placental circulation, we investigated the levels of BH4 in placental extracellular vesicles isolated from perfused placentas obtained from women with or without hypertension in pregnancy, a model system previously demonstrated to reflect alterations in key aspects of placental vascular function, including the levels of eNOS.^19^ We found that BH4 content in placental extracellular vesicles from perfusion of placentas from HTP was significantly lower than those in placental extracellular vesicles from healthy pregnancies (Figure 5F).

### Knockdown of Gch1 Reduces GTPCH Protein and BH4 Levels and Impairs Endothelial Tube Formation in Endothelial Cells

We next tested the causal role of *Gch1* and BH4 in endothelial cell tube formation using small interfering RNA (siRNA) knockdown of *Gch1* in the mouse sEND.1 endothelial cell line that has been widely studied as a model system for eNOS regulation by *Gch1* and BH4.^11,21^ We found that *Gch1*-specific siRNA substantially decreased GTPCH protein levels (Figure 5A) with a corresponding ≈90% reduction in intracellular BH4 levels, compared with nonspecific siRNA controls (Figure 5B). The ratio of BH4 relative to oxidized biopterin species (BH4:BH2+B) was also significantly reduced in cells treated with *Gch1*-specific siRNA (Figure 5B). Consistent with the earlier findings in HUVECs, *Gch1* knockdown impaired endothelial cell tube formation (Figure 5C and 5D), whereas incubation with sepiapterin, which leads to intracellular BH4 synthesis via the pterin salvage pathway, increased BH4 levels and fully restored tube formation (Figure 5C and 5D). Thus, *Gch1* is required for normal BH4 biosynthesis, eNOS activity, and tube formation in cultured endothelial cells, supporting the notion that the reduction in endothelial cell GTPCH and BH4 observed in endothelial cells from HTP could play a causal role in the pathogenesis of pregnancy-related hypertension.

To further test the relevance of these observations to human maternal endothelial cells, we also knocked down *GCH1* in primary human uterine microvascular endothelial cells. *GCH1* knockdown substantially reduced GTPCH protein expression and BH4 levels in human uterine microvascular endothelial cells (Figure 5E) and resulted in a significant reduction in endothelial cell growth in tube formation assays (Figure 5F).

Taken together, these observations indicate that endothelial cell *GCH1* and BH4 regulate endothelial cell growth and tube formation and that reduced endothelial cell BH4 is a feature of pregnancy-induced hypertension and is associated with loss of endothelial cell NOS activity.

### Supplementation With BH4 Fails to Prevents Pregnancy-Induced Hypertension and Fetal Growth Restriction in Gch1^fl/fl^Tie2cre Mice but Is Rescued by the Reduced Folate, 5-Methyltetrahydrofolate

**Figure 6.**
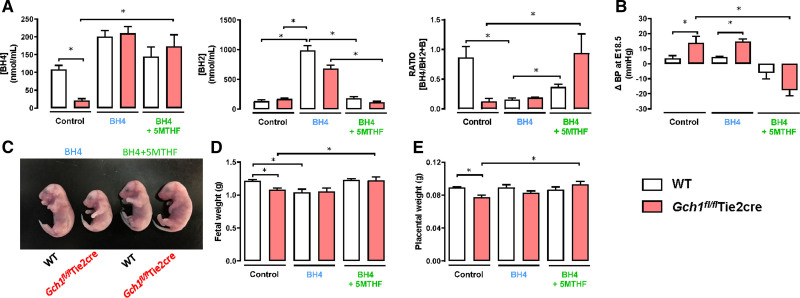
**Supplementation of tetrahydrobiopterin (BH4) and 5-methyltetrahydrofolate (5-MTHF) rescues pregnant-induced hypertension and fetal growth restriction in pregnant mice with endothelial cell BH4 deficiency**. *Gch1*^*fl/fl*^Tie2cre and wild-type (WT) mice were treated with either oral BH4 (200 mg [kg·day], supplemented in chow), or oral BH4 (200 mg [kg·day]) with 5-MTHF (15 mg [kg·day]) or control diet for 3 d before timed-matings, and throughout the subsequent pregnancies. **A**, High-performance liquid chromatography (HPLC) analysis of BH4, dihydrobiopterin (BH2), and BH4:BH2+B ratio in plasma from WT and *Gch1*^*fl/fl*^Tie2cre mice treated with BH4 alone or BH4 with 5-MTHF or control at E18.5 d of gestation (*P<0.05, n=5–7 animals per group). **B**, Change in systolic blood pressure (BP) in WT and *Gch1*^*fl/fl*^Tie2cre mice treated with BH4 alone or BH4 with 5-MTHF or control diet before and at 18.5 d of gestation was measured by noninvasive tail-cuff (*P<0.05, n=5–7 animals per group). **C–E**, Fetal and placental weight determined at E18.5 of gestation (*P<0.05, n=32–43 fetuses from 5 to 7 litters per group). B indicates biopterins.

We reasoned that the consequences of endothelial cell BH4 deficiency in pregnancy might be prevented by BH4 supplementation and that this may have translational therapeutic potential. We treated both *Gch1*^*fl/fl*^Tie2cre and WT mice with chow supplemented with BH4 for 3 days before timed-matings and throughout their subsequent pregnancies. We found that BH4 supplementation increased plasma BH4 levels but was associated with similar or even larger increases in the oxidized species, BH2, with a corresponding reduction in the BH4/BH2+B ratio (Figure 6A and Figure S12), as we have previously observed in patients with established vascular disease.^22^ There were no beneficial effects of oral BH4 supplementation in *Gch1*^*fl/fl*^Tie2cre mice on either BP or fetal growth restriction (Figure 6B through 6E).

The enzyme DHFR (dihydrofolate reductase) reduces dihydrofolate to the fully reduced folate, tetrahydrofolate, and can also reduce oxidized BH2 to regenerate BH4. Accordingly, we hypothesized that coadministration of the fully reduced folate, 5-methyltetrahydrofolate (5-MTHF) may augment vascular BH4 levels.^23^ Coadministration of the fully reduced folate, 5-MTHF led to a striking restoration of BH4 levels in *Gch1*^*fl/fl*^ Tie2cre mice, without the significant elevation of BH2 levels that were observed with BH4 treatment alone. Furthermore, the combination of BH4 and 5-MTHF prevented the increased BP and fetal growth restriction (Figure 6B through 6E).

## Discussion

We have discovered a new role for endothelial cell *Gch1* and BH4 in the vascular adaptation and BP responses to pregnancy, with translational potential for treating the global health challenges of pregnancy-related hypertension, placental insufficiency, and fetal growth restriction. The key findings are (1) selective deficiency of maternal endothelial cell BH4, by targeted *Gch1* deletion in pregnant mice, is sufficient to cause progressive hypertension during pregnancy, impaired uteroplacental vascular remodeling, and fetal growth restriction; (2) pregnancy-induced hypertension is associated with reduced endothelial cell BH4 levels, eNOS activity, and impaired endothelial tube formation, mediated by reduced levels of the BH4 biosynthetic enzyme, GTPCH, encoded by *GCH1*; (3) oral supplementation of BH4 and 5-MTHF, but not BH4 alone, prevents pregnancy-induced hypertension and fetal growth retardation in mice with endothelial cell BH4 deficiency. Taken together, these findings identify a novel role for endothelial cell *Gch1* and BH4 biosynthesis in vascular adaptation and BP regulation to pregnancy. Targeting endothelial cell *Gch1* and BH4 biosynthesis may provide a novel therapeutic target for the prevention and treatment of pregnancy-related hypertension, preeclampsia, and fetal growth retardation.

Although endothelial cell function and NO-mediated vascular remodeling have been previously implicated in pregnancy-related hypertension, such as preeclampsia, the mechanisms, and therapeutic potential have not been elucidated. BH4 bioavailability is a key determinant of eNOS function and NO production^21^ and deficient endothelial cell BH4 biosynthesis, or loss of BH4 by oxidation, leads to eNOS uncoupling, vascular dysfunction, and hypertension. The observation that hypertensive pregnancy is associated with increased circulating levels of BH4 in plasma, but deficient BH4 levels and eNOS function in endothelial cells, is consistent with the known compartmentalization of biopterins between plasma and tissues. The discordance between plasma and vascular BH4 levels is also consistent with the notion of hypertensive pregnancy as a systemic inflammatory state with increased oxidative stress, as we have observed in other cardiovascular disease states, such as diabetes, which is in turn associated with a higher incidence of hypertensive pregnancy.^13,24^

Pregnancy requires sufficient remodeling of both the conduit uterine arteries and the placental resistance vessels to accommodate the ≈10-fold increase in uterine blood flow, and increased cardiac output, without an increase in systemic arterial BP. The critical role of endothelial NO in mediating both functional and structural remodeling is highlighted by the augmented endothelial-dependent vasodilatations and luminal enlargement in uterine arteries from pregnant mice compared with nonpregnant WT mice. eNOS protein and mRNA are increased in uterine arteries in pregnancy,^9,25^ whereas iNOS (inducible NO synthase) and nNOS (neuronal NO synthase) levels remain unchanged.^26^ Acute administration of the NOS inhibitor NG-monomethyl-L-arginine acetate reduced forearm blood flow in pregnant women compared with nonpregnant controls,^27,28^ and L-NG-Nitro arginine methyl ester treatment during pregnancy decreased uterine blood flow in pregnant sheep and rats.^29,30^ A limitation of studies investigating the importance of maternal endothelial cell function in pregnancy is limited by the lack of availability of maternal endothelial cells, for which HUVECs are typically substituted as a model cell culture system. However, HUVECs are fetal in origin and some investigators have sought to validate findings in HUVECs with studies in ovine uterine arteries and endothelial cells.^7^ We used the novel model system of extracellular vesicles obtained from ex vivo perfusion of placentas from women with either normal pregnancies or HTP, these extracellular vesicles are released into the maternal circulation throughout pregnancy. We also reproduced the effects of *GCH1* knockdown, observed in human uterine microvascular endothelial cells, in human primary myometrial endothelial cells, in support of the notion that reduced endothelial cell *GCH1* and BH4 are features of pregnancy-related hypertension, and have effects on endothelial cell function and growth.

We have previously reported that mice with endothelial cell *Gch1* deletion have a small increase in BP, typically ≈7 mm Hg, that we also observed in female *Gch1*^*fl/fl*^Tie2cre mice, before pregnancy. However, we showed that this small increase in prepregnancy BP is not sufficient to explain the pregnancy-associated increase in BP. First, we found that the increase in BP in individual mice was not dependent upon prepregnancy BP. Second, we studied mice with heterozygous *Gch1* deletion in endothelial cells and found that these mice, despite a ≈50% reduction in endothelial BH4 levels, had normal prepregnancy BP and yet still developed hypertension in late pregnancy. Importantly, the changes in pregnancy in endothelial cell *Gch1* deficient mice were entirely dependent upon maternal endothelial cell *Gch1*, not fetal *Gch1*, because WT mice mated with *Gch1*^*fl/fl*^Tie2cre male mice, resulting in litters of the same 1:1 ratio of WT and *Gch1*^*fl/fl*^Tie2cre offspring, showed no hypertension or fetal growth restriction.

The clinical definitions and classification of hypertensive disorders in pregnancy include chronic hypertension that continues or worsens in pregnancy, gestational hypertension, and preeclampsia.^31^ The degree of prepregnancy BP elevation in *Gch1*^*fl/fl*^Tie2cre mice is very modest so would not be considered as a model of chronic hypertension. Nevertheless, chronic hypertension is an important cause of maternal and fetal morbidity and increases the likelihood of progression to preeclampsia. The *Gch1*^*fl/fl*^Tie2cre mouse may be better considered to represent a model of gestational hypertension (ie, hypertension arising in pregnant women, after 20 weeks’ gestation, in the absence of proteinuria and without biochemical or hematological abnormalities), although gestational hypertension does not usually result in fetal growth restriction, as observed in the *Gch1*^*fl/fl*^Tie2cre mouse, that is more usually a consequence of preeclampsia. Indeed, more recent definitions recognize that preeclampsia does not require the presence of proteinuria or renal dysfunction, so in this regard, the pregnancy phenotype in the *Gch1*^*fl/fl*^Tie2cre mouse meets the the International Society for the Study of Hypertension in Pregnancy 2018 definition of preeclampsia,^31^ based on a rise in BP to >140/90 mm Hg after the midpoint of gestation, associated with evidence of fetal growth restriction.

Regarding the mechanism of BH4 in the vascular adaptation in pregnancy, it is well known that NO is a key mediator of vascular adaptation. In pregnancy, increased uterine artery caliber is associated with both enhanced eNOS expression and activity, and NO. Increases in uterine artery blood flow and shear stress enhance eNOS expression and this may contribute to structural arterial remodeling.^32,33^ Indeed, we found that deficiency in endothelial cell BH4 biosynthesis leads to fundamental changes in uterine artery endothelial function and remodeling during pregnancy. The failure of functional remodeling in BH4-deficient *Gch1*^*fl/fl*^Tie2cre uterine arteries in pregnancy demonstrates that the endothelium is a major determinant of both functional and structural vascular remodeling in pregnancy, and confirms a key role for endothelial cell BH4 biosynthesis in eNOS regulation and NO generation during pregnancy. In addition, the structural changes in the vessel wall, particularly in the media, are a very important aspect of the differential response to pregnancy in *Gch*^*fl/fl*^ Tie2cre mice, given that the deletion of *Gch1* is restricted to the endothelium—that is, changes in other cells of the vessel wall are not due to the genetic intervention itself but are due to the functional and structural consequences of endothelial *Gch1* deletion. These changes in uterine endothelial function lead to increased uteroplacental vascular resistance, reduced placental perfusion, and thereby reduced nutrients and oxygen supply to the growing fetuses. Despite reduced fetal and placental weights, litter sizes and onset of parturition were comparable between the WT and *Gch1*^*fl/fl*^Tie2cre mice, indicating that litter sizes or parturition did not contribute to the reduction in fetal and placental weights. This finding is consistent with previous studies in eNOS knockout mice, which demonstrated that eNOS is required in pregnancy to maintain uteroplacental vascular remodeling and normal fetal growth.^34,35^ We now find that specific loss of BH4-mediated eNOS function, termed eNOS coupling is likely to be the major contributor toward vascular dysfunction, inadequate uteroplacental remodeling and hence hypertension observed in pregnant *Gch1*^*fl/fl*^Tie2cre mice. This contrasts with the eNOS knockout mice where all functions of eNOS (ie, both NO and reactive oxygen species generation) are deleted, and where there is marked prepregnancy hypertension, but little or no increase in gestational BP.^36^ Taken together, these findings demonstrate a critical role of endothelial cell BH4 biosynthesis in eNOS regulation in uteroplacental vascular adaptations and BP regulation during pregnancy.

This study reveals a specific requirement for endothelial cell BH4 biosynthesis in BP regulation and uteroplacental remodeling in pregnancy. Plasma BH4 levels were not different between nonpregnant WT and *Gch1*^*fl/fl*^Tie2cre female mice, as previously reported in male mice,^13^ indicating that endothelial cell BH4 synthesis does not play a major role in contributing to circulating BH4. In contrast, plasma BH4 levels were reduced in pregnant WT mice, as previously reported in normal pregnancy in humans.^37^ The additional marked reduction in circulating BH4 in pregnant *Gch1*^*fl/fl*^Tie2cre mice indicates that endothelial cell BH4 makes a significant contribution to circulating BH4 levels during pregnancy and that systemic factors either limit BH4 synthesis or increase BH4 consumption or secretion, for example, by oxidation to BH2 and B. The oxidation of BH4, leading to formation of BH2, B, and potentially other oxidized pterin species is an important pathophysiologic mechanism in other conditions, for example in diabetes and ischemia-reperfusion.

Recycling of BH4 from BH2 can maintain or restore BH4 levels.^14,38^ We found that supplementation of BH4 with the fully reduced folate, 5-MTHF, could restore BH4 levels, and correct the pregnancy phenotype in *Gch1*^*fl/fl*^Tie2cre mice, whereas supplementation with BH4 alone was ineffective, due to BH4 oxidation that has been observed in other studies of oral BH4 administration.^22^ Our study indicates that combination of BH4 with 5-MTHF, or potentially other reducing agents, or other strategies to target BH4 recycling, for example via DHFR, may be effective therapeutic approaches in preeclampsia or other cardiovascular disease states.^21^ Biopterins share structural homology with folates, through the pterin ring. Furthermore, folates and biopterins interact through common metabolic pathways, including reciprocal oxidation-reduction of folates and biopterins via DHFR. Furthermore, MTHF or other fully reduced folates, along with BH4, may have other nonenzymatic effects on cellular reducing capacity and redox state. In particular, 5-MTHF treatment in pregnancy may be an opportunity for clinical translation because folate supplementation is already widely recommended in pregnancy. Whereas clinical trials have shown no benefit of folic acid supplementation in women with preeclampsia, folic acid requires conversion to 5-MTHF via DHFR, so is unable to exert the beneficial redox effects on BH4 observed with 5-MTHF. These novel redox effects of 5-MTHF are likely independent of, or at least in addition to, effects of folates mediated via classical 1-carbon pathways, or via homocysteine lowering.^39,40^ 5-MTHF is available as a nutritional supplement available for human use including during pregnancy. Our previous studies in human vessels showed that 5-MTHF has beneficial effects on endothelial function and vascular superoxide production in human vascular studies, by preventing peroxynitrite-mediated BH4 oxidation and improving eNOS coupling.^23^ Although there is limited data on pregnancy, there have been no reported adverse outcome and it is used in some pregnancy-related supplements.^41,42^

## Perspectives

Inadequate uteroplacental vascular remodeling leads to pregnancy-related hypertension and fetal growth restriction, that are major causes of adverse pregnancy outcomes. These factors have long-term effects on cardiovascular health in both mothers and offspring. Here, we demonstrate that a selective and specific change in endothelial function is sufficient to cause pregnancy-related hypertension, deficient uteroplacental vascular remodeling, and reduced fetal growth, using a targeted mouse model of endothelial cell deficiency of BH4, a required cofactor for NOS activity. We go on to show that human endothelial cells from pregnancies complicated by hypertension have lower levels of BH4 synthesis, associated with reduced endothelial cell NO production. Taken together, the results of this study demonstrate that deficiency in endothelial cell BH4 biosynthesis contributes to pregnancy-induced hypertension and intrauterine growth restriction. These findings suggest a novel role of endothelial cell BH4 biosynthesis in vascular adaptations and BP regulation during pregnancy. Restoration of endothelial cell BH4 with reduced folates identifies a novel therapeutic target to intervene in pregnancy-related hypertension, placental insufficiency, and associated fetal growth restriction. Thus, targeting endothelial cell *GCH1* and BH4 biosynthesis may provide a novel therapeutic target for the prevention and treatment of pregnancy-related hypertension such as preeclampsia.

## Article Information

### Acknowledgments

K.M. Channon conceived the study and designed the experiments, with contributions from N.J. Alp, P. Leeson, and S. Chuaiphichai. Mouse experiments and analyses were done by S. Chuaiphichai with help from G. Douglas, E. McNeill., A.B. Hale, C. Whiteman, Y. Dickinson, M. Appari, and M.J. Crabtree. Micro–computed tomography (CT) studies were undertaken by E.N. Drydale and G. Douglas. Studies in women with preeclampsia, harvested human umbilical vein endothelial cell (HUVECs), and plasma biomarkers analysis were done by G.Z. Yu and A.J. Lewandowski under the supervision of P. Leeson. Tube formation assays were undertaken by S. Chuaiphichai, C.M.J. Tan, and G.Z. Yu. Placental extracellular vesicles studies were undertaken by M. Vatish, W. Zhang, and S. Chuaiphichai. The article was drafted by K.M. Channon and S. Chuaiphichai. All authors discussed the results and had the opportunity to contribute to the article.

### Sources of Funding

This study was supported by a British Heart Foundation (BHF) Programme Grants (RG/12/5/29576 and RG/17/10/32859), BHF Project Grant (PG/19/48/34433), BHF Chair award (CH/16/1/32013), Oxford BHF Centre of Research Excellence (RE/13/1/30181), and the National Institute for Health Research (NIHR) Oxford Biomedical Research Centre.

### Disclosures

None.

## Supplementary Material



## Novelty and Significance

### What Is New?

- We demonstrate that a selective change in endothelial function is sufficient to cause pregnancy-related hypertension, deficient uteroplacental vascular remodeling, and reduced fetal growth, using a targeted mouse model of endothelial cell deficiency of tetrahydrobiopterin.
- We also show that human endothelial cells from pregnancies complicated by hypertensive pregnancies have lower levels of tetrahydrobiopterin synthesis, associated with reduced NO production.
- We demonstrate that supplementation of tetrahydrobiopterin (BH4) with the fully reduced folate, 5-methyltetrahydrofolate, could restore BH4 levels, and correct the pregnancy phenotype in *Gch1*^*fl/fl*^Tie2cre mice, whereas supplementation with BH4 alone was ineffective.

### What Is Relevant?

- Inadequate uteroplacental vascular remodeling leads to pregnancy-related hypertension and fetal growth restriction, which are major causes of adverse pregnancy outcomes, affecting ≈7% of all pregnancies worldwide.
- Emerging evidence suggests that these factors have long-term effects on cardiovascular health in both mothers and offspring.
- This study identifies a novel role of endothelial cell BH4 biosynthesis in vascular adaptations and blood pressure regulation during pregnancy.

### Summary

Using multiple complementary in vitro, ex vivo, and in vivo translational models, we demonstrate that deficiency in endothelial cell BH4 biosynthesis contributes to pregnancy-induced hypertension and intrauterine growth restriction. These findings suggest a novel role of endothelial cell BH4 biosynthesis in vascular adaptations and blood pressure regulation during pregnancy. Restoration of endothelial cell BH4 with reduced folates identifies a novel therapeutic target for the prevention and treatment of pregnancy-related hypertension such as preeclampsia.
