# Supplementary material for: Endothelial GTPCH (GTP Cyclohydrolase 1) and Tetrahydrobiopterin Regulate Gestational Blood Pressure, Uteroplacental Remodeling, and Fetal Growth
Source: Hypertension. 2021 Oct 25;78(6):1871–84. doi: 10.1161/HYPERTENSIONAHA.120.17646 (PMC8577301; doi:10.1161/HYPERTENSIONAHA.120.17646)
Supplement: Supplementary file 1 [file hyp-78-1871-s001.pdf]

# Endothelial GTP Cyclohydrolase and Tetrahydrobiopterin Regulate Gestational Blood Pressure, Uteroplacental Remodeling and Fetal Growth

**Running Title:** *Chuaiphichai et al*; *Gch1 and BH4 in Pregnancy*

Surawee Chuaiphichai[1*], Grace Z. Yu[2], Cheryl M.J. Tan[2], Christopher Whiteman[1], Gillian Douglas[1] , Yasmin Dickinson[1], Edward N. Drydale[1], Mahesh Appari[1], Wei Zhang[3] Mark J. Crabtree[1], Eileen McNeill[1], Ashley B. Hale[1], Adam J. Lewandowski[2], Nicholas J. Alp[1], Manu Vatish[3, 4], Paul Leeson[2], Keith M. Channon[1, 4*]

[1]Division of Cardiovascular Medicine, British Heart Foundation Centre of Research Excellence, Radcliffe Department of Medicine, University of Oxford, Oxford, OX3 9DU, UK

[2]Oxford Cardiovascular Clinical Research Facility, Division of Cardiovascular Medicine, Radcliffe Department of Medicine, University of Oxford, Oxford OX3 9DU, UK

[3]Nuffield Department of Women's and Reproductive Health, University of Oxford, Oxford OX3 9DU, UK

[4]National Institute for Health Research (NIHR) Oxford Biomedical Research Centre, Oxford University Hospitals NHS Foundation Trust, John Radcliffe Hospital, Oxford OX3 9DU, UK

[*]Corresponding authors:

Surawee Chuaiphichai, DPhil
Division of Cardiovascular Medicine
British Heart Foundation Centre of Research Excellence,
Radcliffe Department of Medicine,
University of Oxford,
Oxford, OX3 9DU, UK
Tel: +44(0)1865 287662
e-mail: surawee.chuaiphichai@cardiov.ox.ac.uk

Keith M.Channon, FMedSci FRCP, MD
Division of Cardiovascular Medicine
British Heart Foundation Centre of Research Excellence,
Radcliffe Department of Medicine,
University of Oxford,
Oxford, OX3 9DU, UK
Tel: +44(0)1865 572783
e-mail: keith.channon@cardiov.ox.ac.uk

## DATA SUPPLEMENT

### HUVEC Isolation, HutMECS Culture and Matrigel Assay

Umbilical cords were collected at birth and human umbilical vein endothelial cells (HUVECs) were isolated and stored in liquid nitrogen according to standard operating procedures within a research tissue bank (Oxford Cardiovascular Tissue Bioresource; ethical approval 09/H0606/68, 07/H0606/148 and 11/SC/0230). For the purpose of the current study, HUVECs were identified from normotensive pregnancies and pregnancy-induced hypertension, matched for maternal age and gestation. HUVECs and human uterine microvascular endothelial cells (HutMECs; Cat C-12295, Promocell) were cultured in EBM-2 (endothelial basal medium) with bullet kit as recommended (Cat CC-3162, Lonza). All cell cultures were maintained in humidified 5% $CO_2$ at 37°C. Primary HUVEC and HutMEC cells, obtained between passages 1-3 were and passages 4-6, respectively, were used for all experiments. sEnd.1 endothelial cells were grown in Dulbecco's modified Eagle's medium (Invitrogen) supplemented with glutamine (2 mmol/liter), penicillin (100 units/ml), and streptomycin (0.1 mg/ml).

To assess tube formation ability of endothelial cells, a 96-well plate was evenly coated with 50µl of growth factor-reduced Matrigel (BD Biosciences, UK). Endothelial cells were plated at a density of $1x10^4$ cells per well. The plate was incubated at 37˚C for 16 hours before photomicroscopy. Each sample was replicated in triplicate and the image of each well was taken at x4 magnification using a Nikon Eclipse TE2000-U microscope (Nikon Ltd, London, UK). Images obtained from Matrigel assay were adjusted for mean brightness using acquisition software to control the bright field illumination of the microscope (Simple PCI version 6.6.0.0; Hamamatsu Corporation, Sewickley, PA). Images were saved as TIFF files, and tube formation analysed using AngioSys 1.0 (TCS Cell Works, UK). Image threshold was adjusted based on the intensity values of the monochrome image and each image then skeletonized to reduce to one pixel wide. A line was drawn over each tubule and each branch point marked with a dot. The total length of lines was quantified in pixels (then converted to micrometers) and total number of branch points was recorded.

### *GCH1* Knockdown by RNA Interference

*GCH1*-specific, ON-TARGETplus SMARTpool siRNA was purchased from Dharmacon Thermo Scientific. 24 h prior to transfection, the cells were seeded into 6-well plates. The cells were then transfected with GCH1-specific siRNA (100 nmol/liter), GAPDH-positive (100 nmol/liter) or nonspecific pooled duplex negative control siRNA (100 nmol/liter). The cells were cultured for 72 h, and gene silencing was detected by analysis of GTPCH protein expression by Western blotting using GTPCH-specific antibodies (a gift from S.Gross, Cornell University New York), in cells using standard protocols.

### Maternal blood sample analysis

Plasma circulating pro-angiogenic and anti-angiogenic factors were quantified with commercial enzyme-linked immunosorbent assays (ELISAs). All samples, standards, and controls were plated in duplicate. Optical density of each well was measured at 450nm using a FLUOstar Omega microplate reader (BMG Labtech, KBioScience, USA). Data was analyzed using Omega Data Analysis software. Duplicate readings for each standard, control, and sample were averaged, and the average zero standard optical density was subtracted. Standard curves were created by generating a four-parameter logistic curve-fit. The coefficients of variation for sFlt-1 was 4.5% with a SD of 1.9%, and for sEng it was 4.1% with a SD of 1.6%.

### Blood pressure measurement by implantable telemetry

Non-pregnant female $Gch1^{fl/fl}$Tie2cre and $Gch1^{fl/fl}$ (wild-type) mice (8–10-week-old) underwent thoracic aortic implantation of telemeters (PAC10 radiotelemeters; DSI, Transoma Medical Inc.). Briefly, telemeter catheters were implanted in the left carotid artery with the body of the telemeter placed in a subcutaneous pocket equidistant from the fore and hind paw. The wound was then closed with 4.0 vicryl. Post-operatively, mice were held in a recovery chamber at 37° until mobile and subsequently moved to a recovery cabinet at 28° for a further 4 h. After 14 days of recovery in home cages (placed on top of telemetry receivers), telemeters were magnetically activated, and baseline mean arterial blood pressure (MAP) was recorded continuously for 5 days (with sampling every 1 minutes for 10-second intervals). Pregnancy was achieved by mating either female $Gch1^{fl/fl}$Tie2cre or $Gch1^{fl/fl}$ (wild-type) females with a $Gch1^{fl/fl}$ male. To evaluate the gestation day, vaginal plugs were checked for the following morning, taken as the 0.5 day of gestation (E0.5). MAP was recorded continuously throughout the pregnancy until E18.5 day of gestation.

### Blood pressure measurement by tail-cuff plethysmography

Systolic blood pressure and heart rate was determined using a computerized tail-cuff system (Visitech, USA) in conscious mice. Experiments were performed between the hours of 8 and 12 am. The animal tails were passed through a cylindrical latex tail-cuff and taped down to reduce movement. Twenty readings were taken per mouse of which the first 5 readings were discarded. The remaining 15 readings were used to calculate the mean systolic blood pressure and heart rate in each mouse.

### Analysis of NO Synthesis by eNOS

NO synthesis by eNOS was assessed by conversion of $^{14}C$ L-arginine to citrulline, in the presence and absence of N-monomethyl-L-arginine (1 mM, Sigma). Briefly, HUVES were

incubated for 4 hours at 37°C in 200 µl Krebs-HEPES buffer containing $^{14}C$ L-arginine (2 µl of 50 µCi/mL). Samples were run on a SCX 300 cation-exchange HPLC column (Sigma) with online scintillation detection. Background signals were corrected from samples with $^{14}C$ L-arginine alone without cells.

**Micro CT imaging**

Placentas were imaged using a SkyScan 1172 micro-CT (Bruker). The placentas were mounted in 1.5% agarose in a sealed sample holder. X-ray images were generated at a voltage of 45kv and a current of 218µA, with no filter applied. Scanning resolution was set at 2.5µM per pixel. A virtual image stack generated using NRecon software (Bruker). The image stack was downsized to a resolution of 10µM per pixel. 3D reconstructions were generated using AMIRA software (version 5.5.0).

# Figure S1

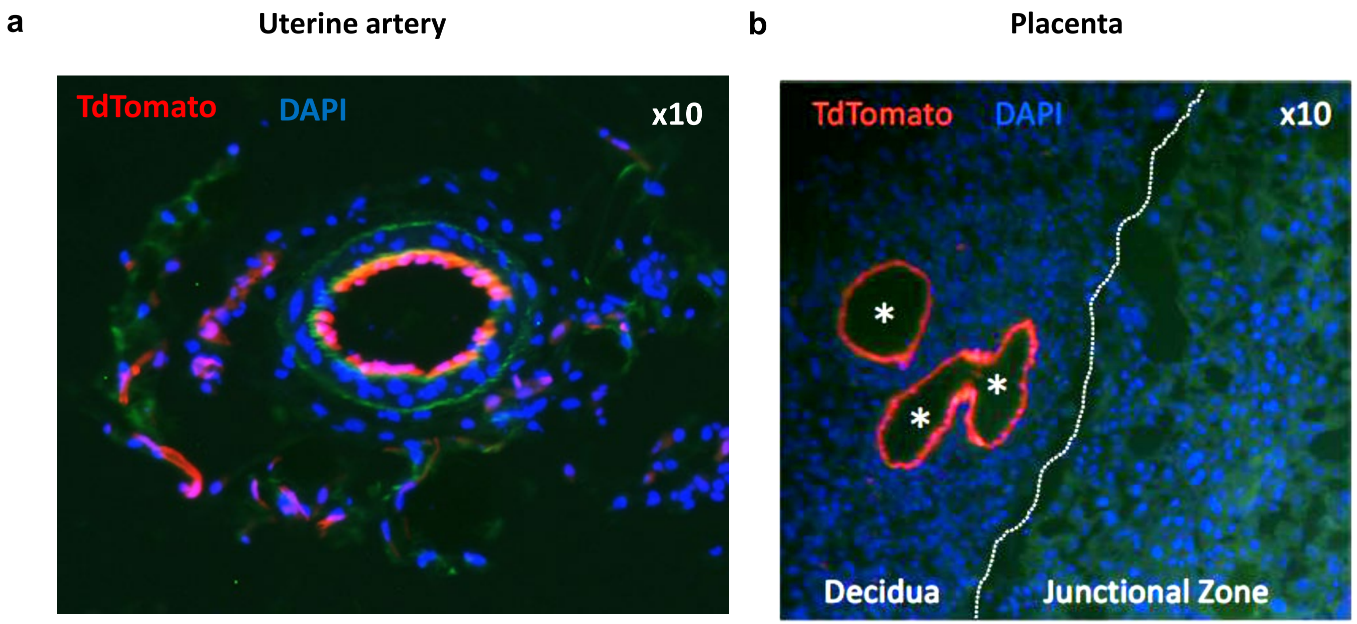


**Figure S1: Endothelial cell-specific floxed allele excision in pregnant Tie2cre mouse uterine artery and placenta**

Tie2cre mice were crossed with floxed TdTomato reporter mice. Female Tie2cre/TdTomato mice underwent timed matings with WT male mice. Uterine arteries and placental tissues were harvested at E18.5 day of gestation for fluorescence microscopy.

Red Tdt fluorescence highlights endothelial cells in **(a)** uterine arteries and **(b)** decidual spiral arteries (*), respectively. Nuclei are stained blue with DAPI.

# Figure S2

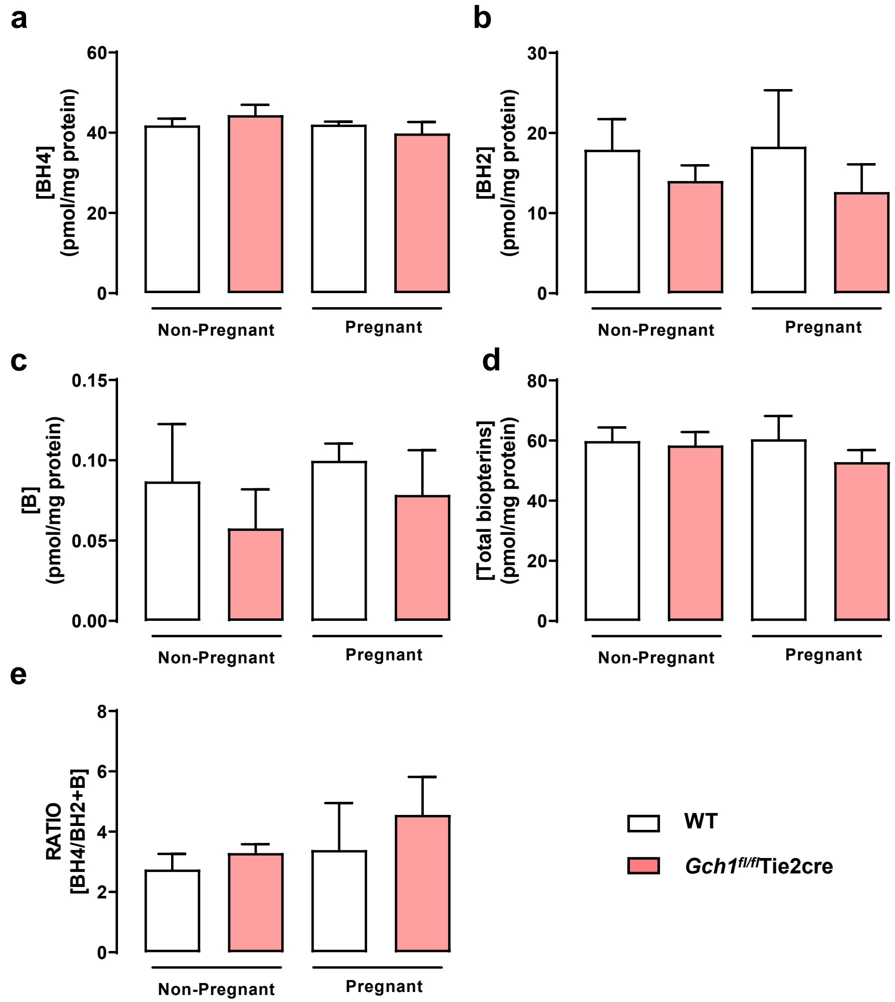


**Figure S2. Liver Biopterin Levels in Wild Type and *Gch1*$^{fl/fl}$Tie2cre Mice in Pregnancy**

(**a - e**) Levels of BH4, BH2, B were measured by HPLC in liver tissue homogenates obtained from wild type (WT) and *Gch1*$^{fl/fl}$Tie2cre mice, both non-pregnant and at the end of pregnancy. Total Biopterins (BH4+BH2+B) and BH4/(BH2+B) ratio were calculated (n=6 animals per group).

# Figure S3

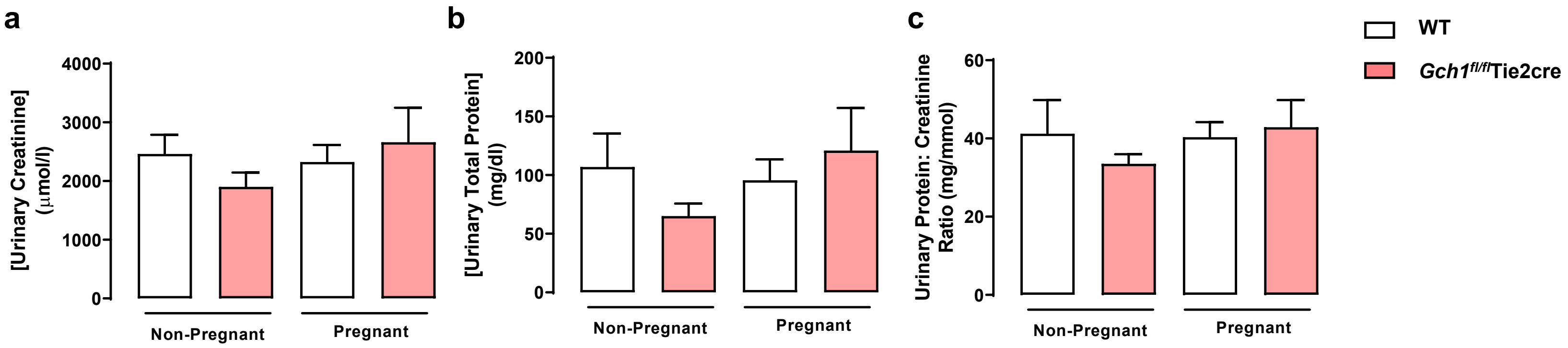


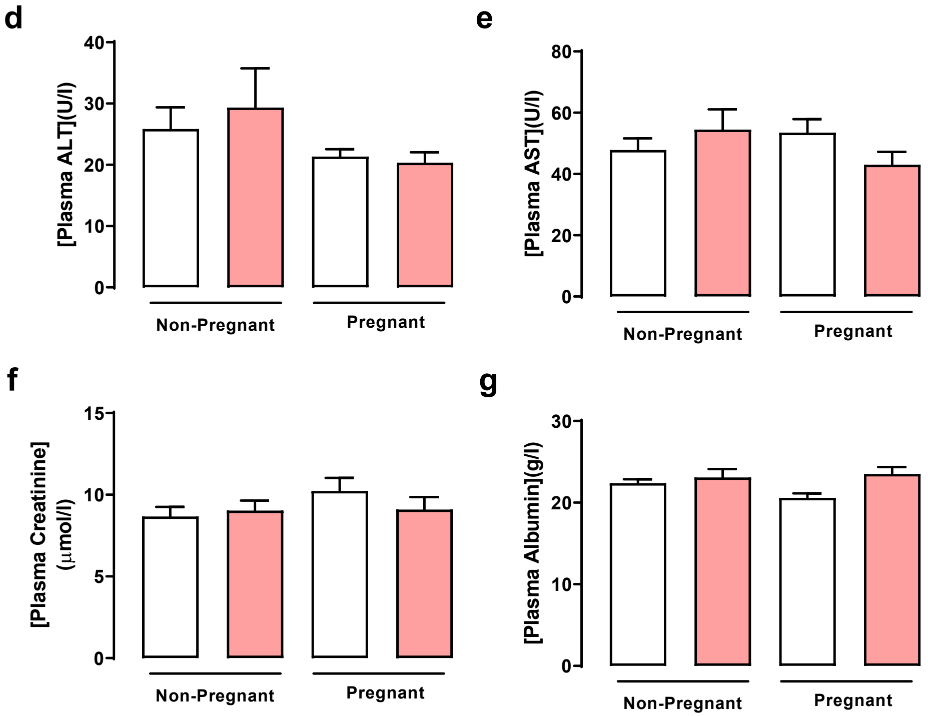


**Figure S3. Urinary Protein and Plasma Protein and Liver enzyme Levels in Wild Type and *Gch1*$^{fl/fl}$Tie2cre Mice in Pregnancy**

(**a - c**) Levels of creatinine and total protein were measured by clinical chemistry analyser in urine obtained from wild type (WT) and *Gch1*$^{fl/fl}$Tie2cre mice, both non-pregnant and at the end of pregnancy. Urinary protein/creatinine ratio were calculated (n= 6 animals per group).
(**d - g**) Levels of alanine transaminase (ALT), Aspartate transaminase (AST), creatinine and albumin were measured by clinical chemistry analyser in plasma obtained from wild type (WT) and *Gch1*$^{fl/fl}$Tie2cre mice, both non-pregnant and at the end of pregnancy (n= 6 animals per group).

# Figure S4

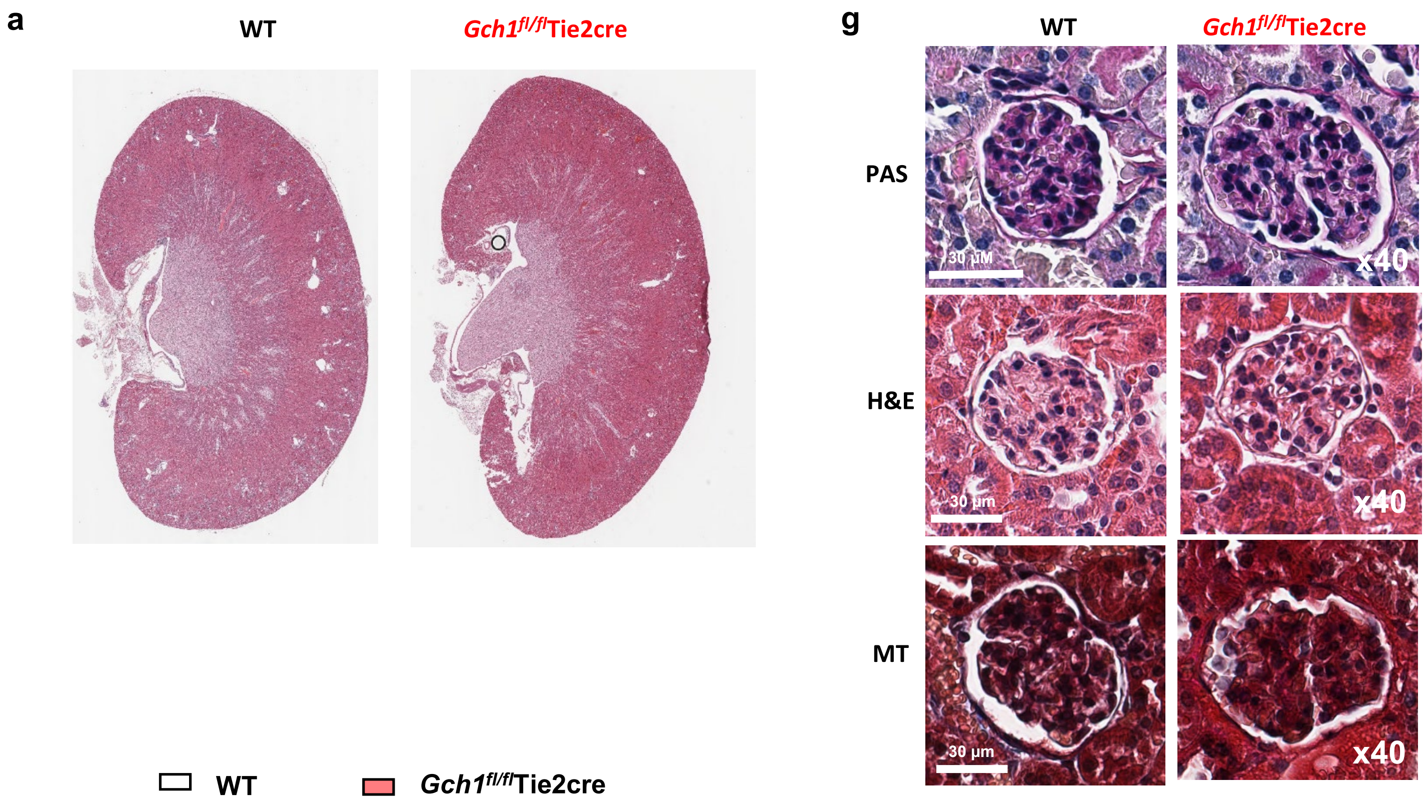


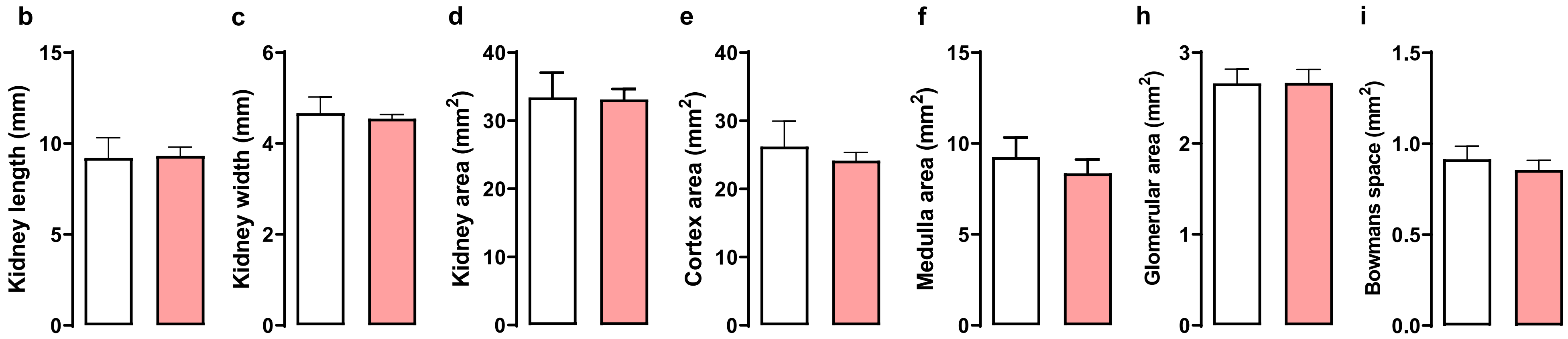


**Figure S4: Renal Histology in *Gch1*$^{fl/fl}$Tie2cre mice**

Kidneys were harvested from pregnant wild type (WT) and *Gch1*$^{fl/fl}$Tie2cre mice, fixed and processed for histology. Sections were stained with periodic acid-Schiff (PAS), hematoxylin and eosin (H&E) or Masson trichrome stains. Dimensions and areas were measured using Image J.

(a) Macroscopic images of kidney saggital cross sections from WT and *Gch1*$^{fl/fl}$Tie2cre mice.

**(b - f)** Histologic measurements of kidney length, width, total area and cortical and medullary area.

**(g)** Histologic images (x40 magnification, bar = 30um) of renal glomeruli from WT and *Gch1*$^{fl/fl}$Tie2cre mice.

**(h, i)** Quantification of glomerular area and Bowman's space area from multiple glomeruli (n=6 to 7 animals per group).

# Figure S5

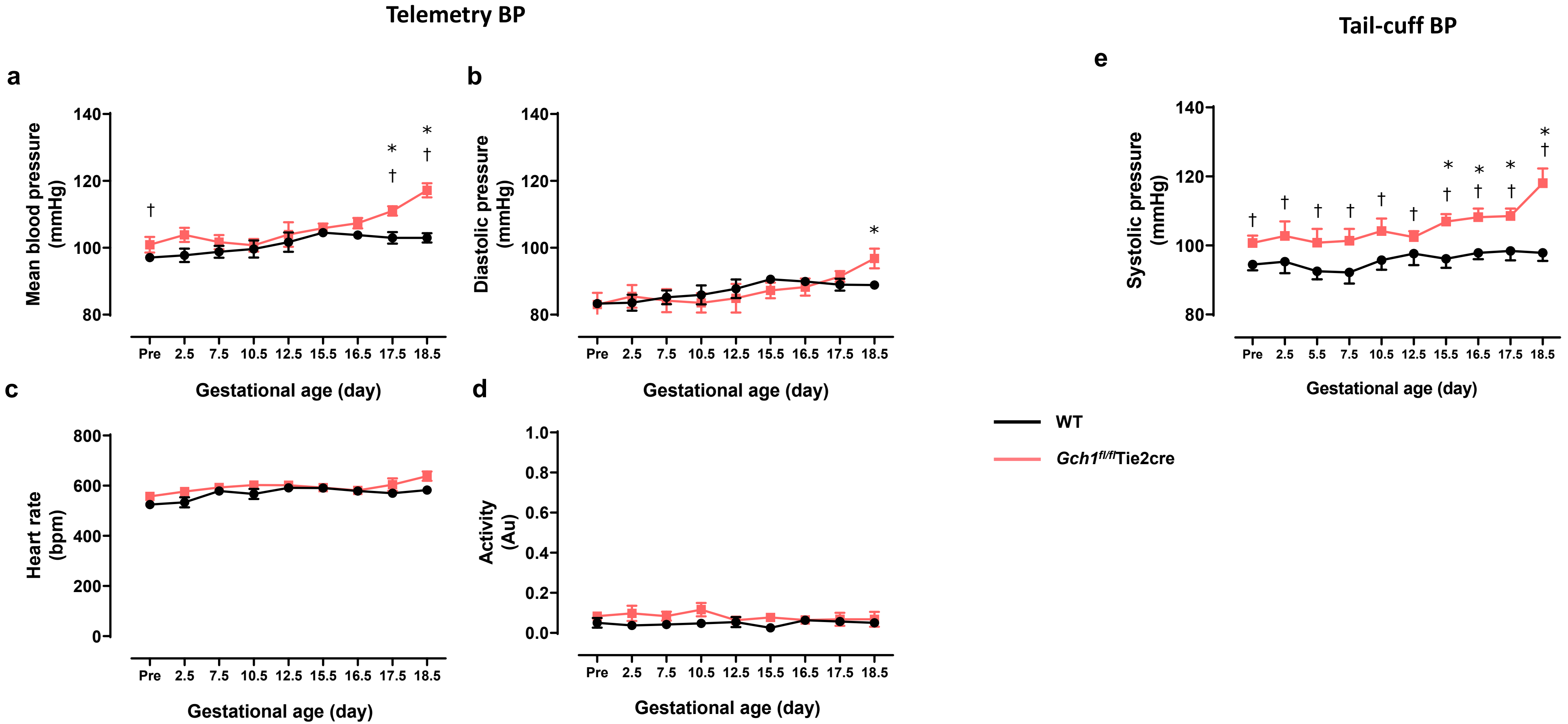


**Figure S5: Telemetry Blood Pressure and tail-cuff blood pressure in Pregnancy in Wild Type and *Gch1*$^{fl/fl}$Tie2cre mice**

Female mice, either wild type (WT) or *Gch1*$^{fl/fl}$ Tie2cre, were mated with *Gch1*$^{fl/fl}$ Tie2cre or WT male mice (to generate genetically matched litters), and blood pressure was measured during pregnancy by blood pressure telemetry (a-d) and tail-cuff method (e).

**(a-b)** Mean arterial pressure and diastolic pressure were measured during pregnancy. Both systolic and mean blood pressure at E18.5 days of gestation in *Gch1*$^{fl/fl}$ Tie2cre female mice were significantly higher than those in the wild-type littermate controls. (n=5 and n=7, respectively) † Denotes p<0.05 vs. WT; * denotes p<0.05 vs. baseline blood pressure.

**(c-d)** Heart rate and activity throughout pregnancy for *Gch1*$^{fl/fl}$ Tie2cre or WT female mice (n=5 and n=7, respectively).

**(e)** Systolic blood pressure was measured by non-invasive tail-cuff plethysmography in wild-type (*Gch1*$^{fl/fl}$) and *Gch1*$^{fl/fl}$Tie2cre mice before and during pregnancy. († *P<0.05* comparing genotype; **P<0.05* comparing baseline blood pressure; n=7 to 10 animals per group).

# Figure S6

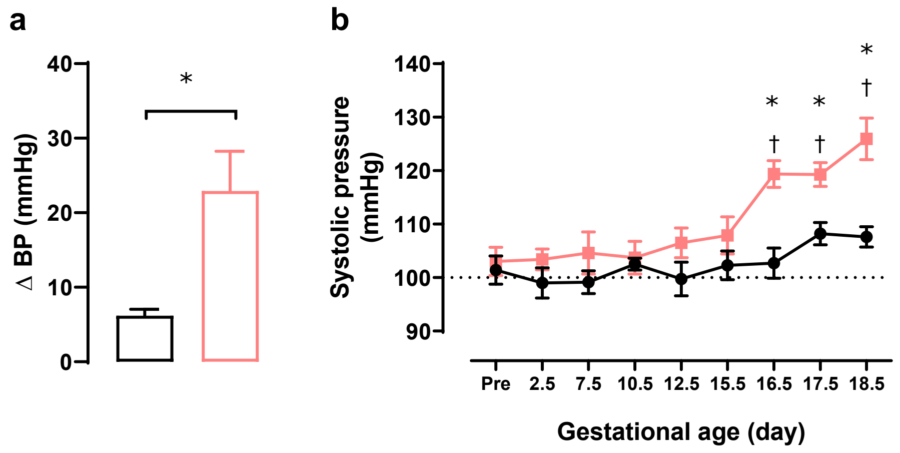


**Figure S6: Blood Pressure Changes in Pregnancy in Wild Type and *Gch1$^{fl/fl}$*Tie2cre mice Matched for Baseline Blood Pressure**

Female mice, either wild type (WT) or *Gch1$^{fl/fl}$* Tie2cre, were mated with *Gch1$^{fl/fl}$* Tie2cre or WT male mice (to generate genetically matched litters), and blood pressure was measured every 3 days during pregnancy by tail cuff plethysmography.

**(a)** Mean change in blood pressure between early pregnancy (e2.5) and late pregnancy (e18.5) for WT or *Gch1$^{fl/fl}$* Tie2cre female mice (n=5 and n=6, respectively)

**(b)** Blood pressure profiles throughout pregnancy for WT or *Gch1$^{fl/fl}$* Tie2cre female mice (n=5 and n=6, respectively) that were selected from the cohort to ensure equal blood pressure at baseline. Even after this baseline covariate adjustment, the increase in BP during pregnancy remained much greater in the *Gch1$^{fl/fl}$* Tie2cre mice compared with WT mice.

* Denotes $p<0.05$ vs. WT; † denotes $p<0.05$ vs. baseline blood pressure.

# Figure S7

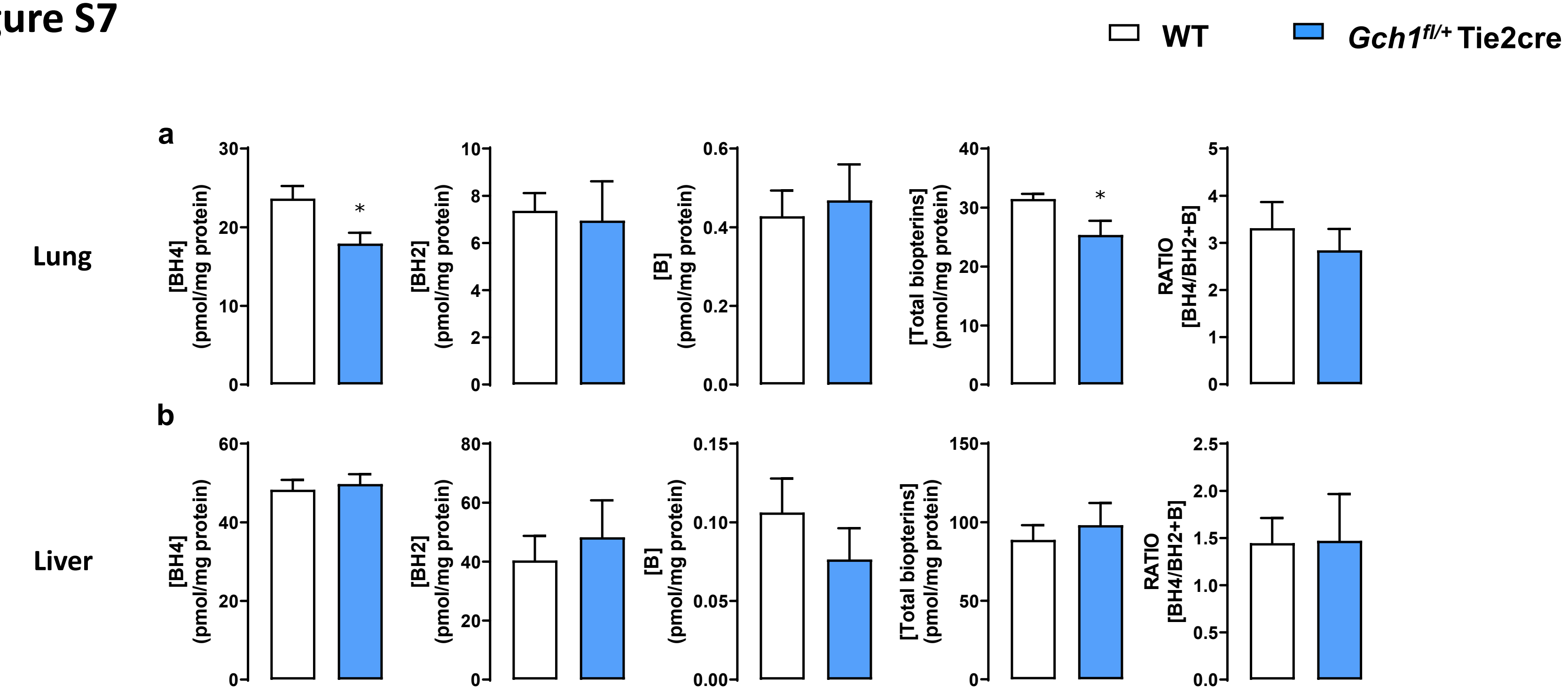


**Figure S7. Biopterin Levels in Mice with Heterozygous Deletion of *Gch1* in Endothelial Cells (i.e. *Gch1$^{fl/+}$* Tie2cre Mice) in Pregnancy.**

Mice with heterozygous deletion of *Gch1* in endothelial cells (i.e. *Gch1$^{fl/+}$* Tie2cre mice) were generated by crossing *Gch1$^{fl/fl}$* Tie2cre mice with WT (i.e. *Gch1$^{+/+}$*) mice. Female *Gch1$^{fl/+}$* Tie2cre mice were mated with WT male mice.

**(a-b)** Levels of BH4, BH2, B were measured by HPLC in tissue homogenates obtained from wild type (WT) and *Gch1$^{fl/+}$* Tie2cre mice, at the end of pregnancy. Total Biopterins (BH4+BH2+B) and BH4/(BH2+B) ratio were calculated. Shown are biopterin levels in lung (**a**), and liver (**b**)

* Denotes $p<0.05$ vs. WT.

# Figure S8

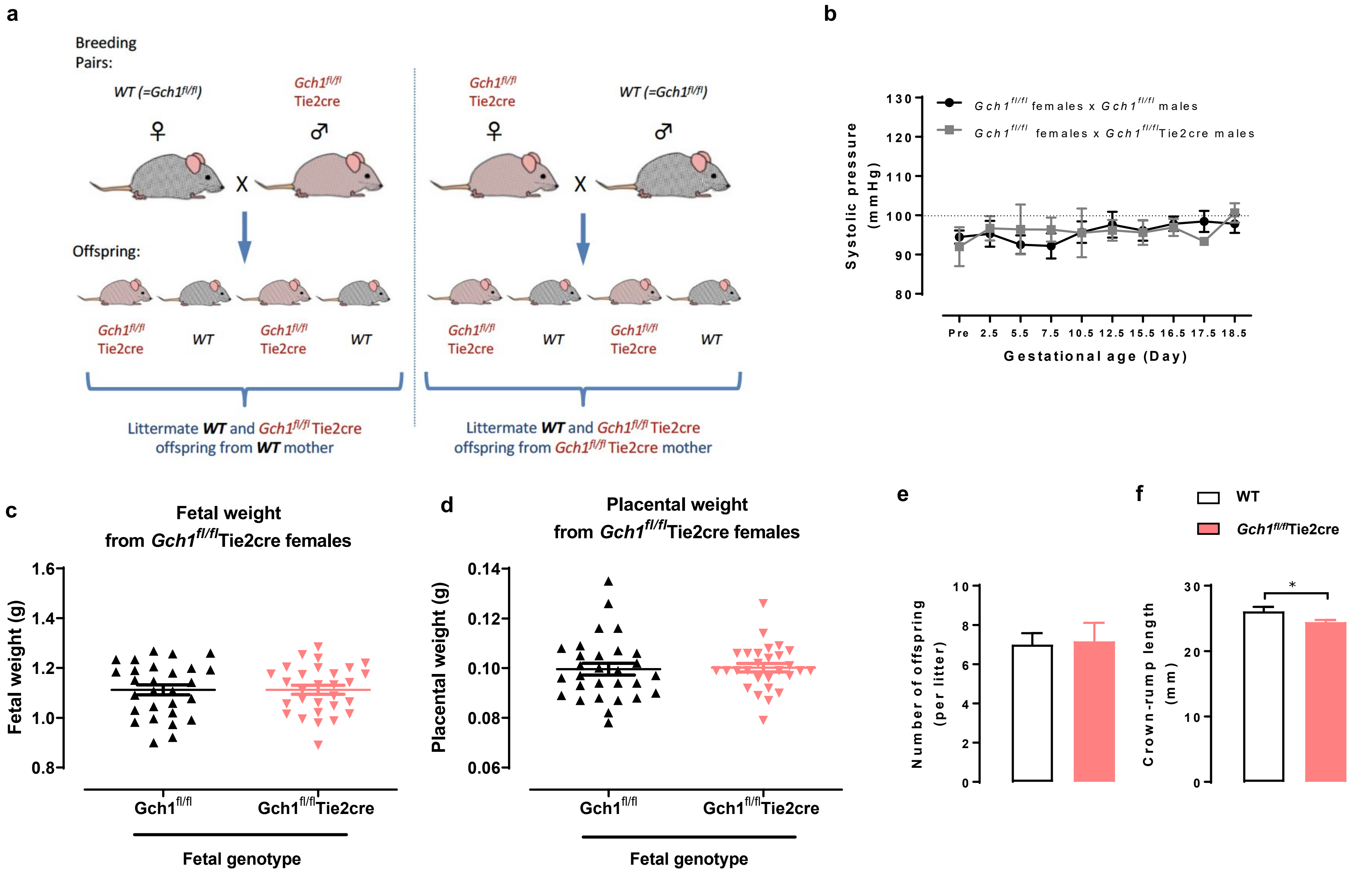


**Figure S8**

**(a)** Schematic of breeding pairs of either female WT (i.e. *Gch1*$^{fl/fl}$) crossed with male *Gch1*$^{fl/fl}$Tie2cre mice, or female *Gch1*$^{fl/fl}$Tie2cre crossed with male WT mice. Both breeding pairs produce the same littermates composed of WT or *Gch1*$^{fl/fl}$Tie2cre offspring in 1:1 ratio, with the key difference being that the genetically matched offspring are either born to a WT mother or a *Gch1*$^{fl/fl}$Tie2cre mother.
**(b)** Systolic blood pressure, measured by non-invasive tail-cuff, in wild-type females mated with either wild-type males or *Gch1*$^{fl/fl}$Tie2cre males before and during pregnancy. (n=7 to 10 animals per group).
**(c and d)** fetal and placental weights from *Gch1*$^{fl/fl}$Tie2cre mothers were determined according to fetus genotypes. No difference in fetal and placental weights between wild-type fetus and *Gch1*$^{fl/fl}$Tie2cre fetus from *Gch1*$^{fl/fl}$Tie2cre mothers.
**(e)** No difference in litter size between wild-type and *Gch1*$^{fl/fl}$Tie2cre mothers at birth.
**(f)** Live pups from wild-type and *Gch1*$^{fl/fl}$Tie2cre mothers were collected and measured at birth.

# Figure S9

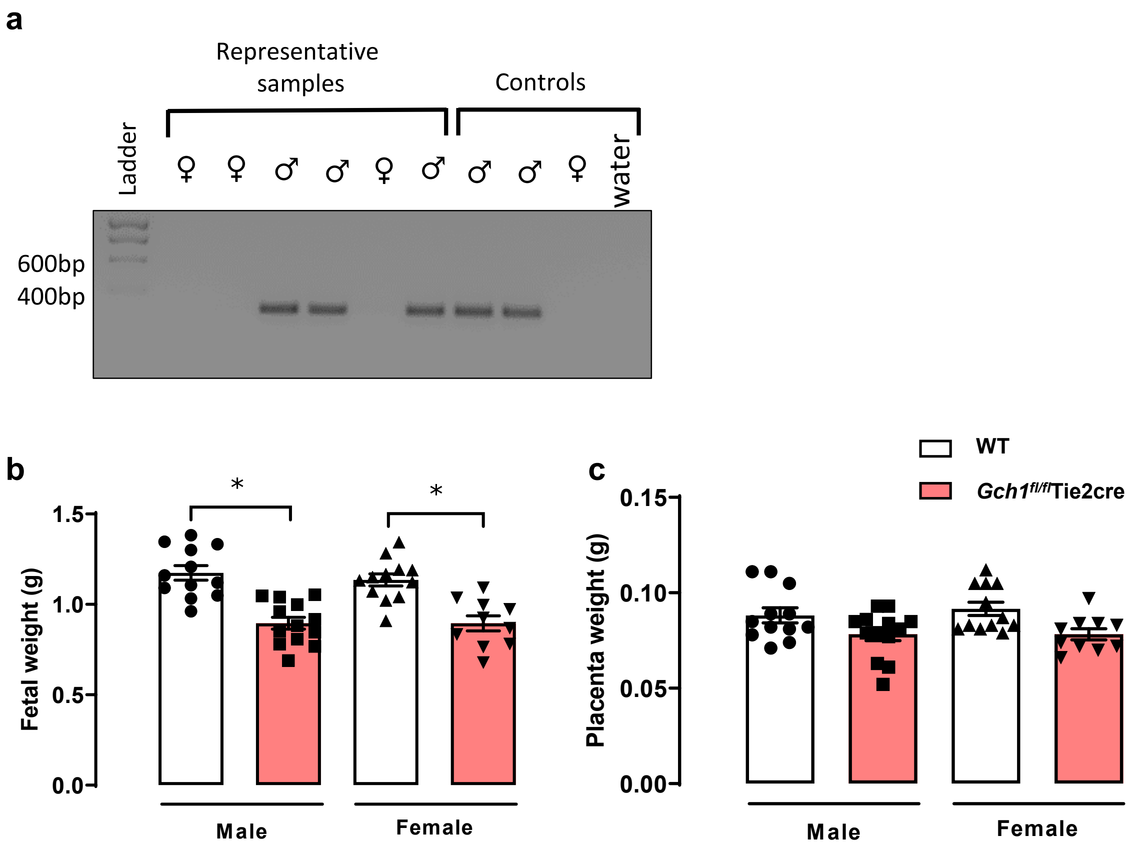


**Figure S9. Fetal and placental weights of male and female fetuses from Wild Type and *Gch1*$^{fl/fl}$Tie2cre Mothers**

**(a)** Representative of sex of the embryos from wild-type and *Gch1*$^{fl/fl}$Tie2cre mothers was genotyped using *Zfy* primer to detect Y chromosome.
**(b - c)** Fetal and placental weights from wild-type and *Gch1*$^{fl/fl}$Tie2cre mothers were collected at E18.5 day of gestation and weighed according to fetus genotypes and genders. Male and female fetuses from pregnant *Gch1*$^{fl/fl}$Tie2cre mothers were significantly smaller compared to wild-type mothers. No male-female difference in the reduction in fetal weight.

(* denotes $P<0.05$; 25 males and 22 females from n= 4 WT mothers and n=4 *Gch1*$^{fl/fl}$Tie2cre mothers).

# Table S1

**Table 1. Characteristics of Cohort (HUVECs)**

| Parameters | Normotensive (n=14) | Hypertensive (n=12) |
|---|---|---|
| **Maternal** | | |
| Maternal age, years | 33.6±2.9 | 35.4±3.8 |
| BMI at booking, $kg/m^2$ | 23.4±2.9 | 30.1±8.2* |
| Smokers, n (%) | 2(14.3) | 4(40) |
| LFT, n (%) | 0(0) | 3(30) |
| Booking sBP, mmHg | 107.1±11.4 (86-126) | 120.6±12.5 (90-130)* |
| Booking dBP, mmHg | 64.4±8.6 (50-83) | 74.9±8.4 (60-84)* |
| Late gestation sBP, mmHg | 108.6±9.1 (90-120) | 122.1±16.6 (102-153)* |
| Late gestation dBP, mmHg | 63.4±8.5 (50-81) | 81.9±12.7 (55-93)*** |
| Highest sBP, mmHg | 119.6±13.7 (98-148) | 157.6±26.0 (145-200)*** |
| Highest dBP, mmHg | 72.9±8.5 (60-87) | 97.5±11.2 (88-135)*** |
| **Fetal** | | |
| Gestational age, weeks | 39.5±2.1 | 36.7±2.4** |
| Males, n (%) | 8(57.1) | 6(54.5) |
| Birthweight, grams | 3390.4±731.8 | 2655.1±802.3* |
| Birthweight z-score | 0.3±0.9 | -0.6±1.0* |
| Head circumference, mm | 173.8±10.2 | 173.9±10.4 |
| Abdominal circumference, mm | 141.8±41.5 | 152.2±16.3 |
| Femur length, mm | 31.9±2.3 | 31.3±3.2 |

Values as Mean±Standard Deviation unless stated otherwise.

sBP systolic blood pressure; dBP diastolic blood pressure; LFT; liver function test. Statistically significant *p*-values are asterisked. * $p<0.05$; ** $p<0.01$; *** $p<0.001$

# Figure S10

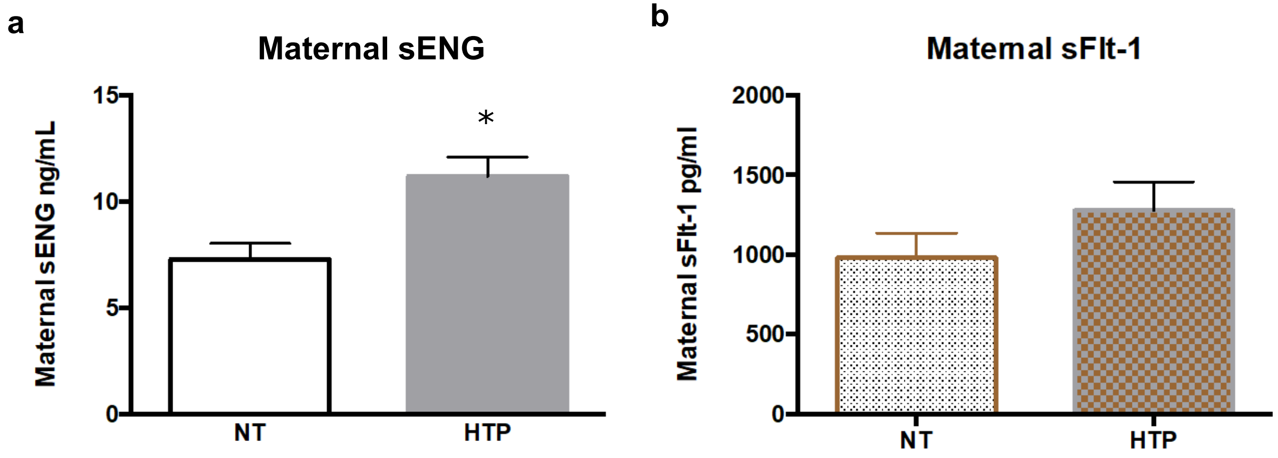


**NT** – Normotensive
**HTP –** Hypertensive Pregnancies

**Figure S10. Plasma Biomarkers in Women with Normotensive (NT) or Hypertensive (HTP) Pregnancies**
(**a and b**) plasma anti-angiogenic markers soluble endoglin (sENG) and soluble fms-like tyrosine kinsase-1 (sFlt-1) were measured by enzyme-linked immunosorbent assay on 5 days after delivery (n=12 to 14 per group). Levels of sENG and sFlt-1 at 5 days post-partum are lower than on the final day of pregnancy, but are closely correlated and remain representative of pregnancy levels (see Yu et al. Hypertension 2016;68:749-59).

# Figure S11

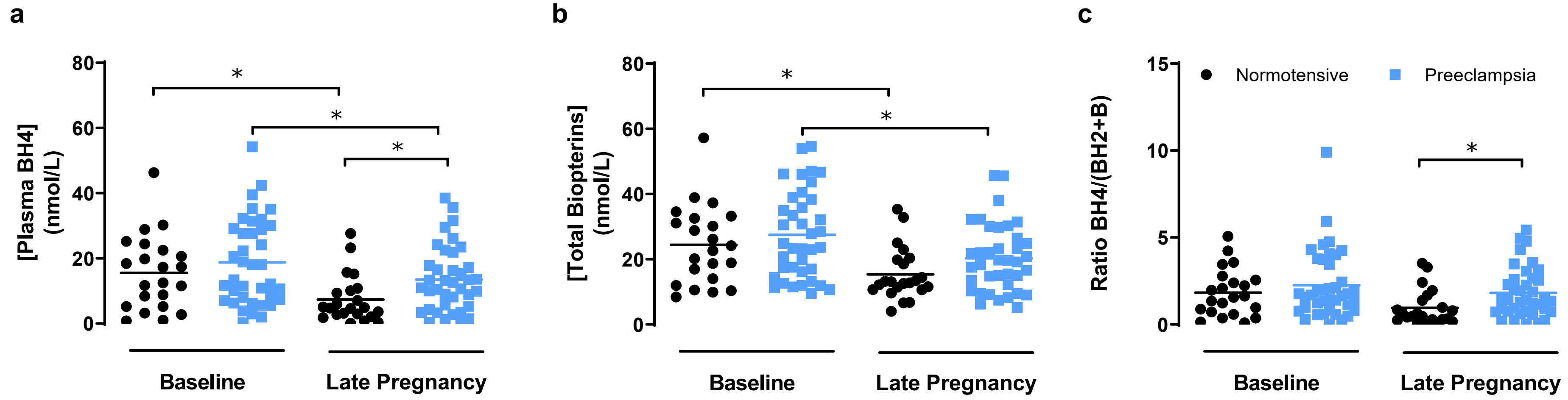


**Figure S11. Plasma BH4 in Women with Normotensive (NT) or Hypertensive (HTP) Pregnancies**

(**a, b and c**) Levels of BH4, total biopterins, and BH4/BH2+B ratio were measured by HPLC in plasma from women with preeclampsia (n=38) and in normotensive controls (n=24) at baseline (3 months after pregnancy) and late pregnancy. Black symbols denote NT, blue symbols denote PE. * denotes $p<0.05$ for PE vs. NT, or late pregnancy vs. early pregnancy.
