# Supplementary figures and images for: Endothelial GTPCH (GTP Cyclohydrolase 1) and Tetrahydrobiopterin Regulate Gestational Blood Pressure, Uteroplacental Remodeling, and Fetal Growth
Source: Hypertension. 2021 Oct 25;78(6):1871–84. doi: 10.1161/HYPERTENSIONAHA.120.17646 (PMC8577301; doi:10.1161/HYPERTENSIONAHA.120.17646)

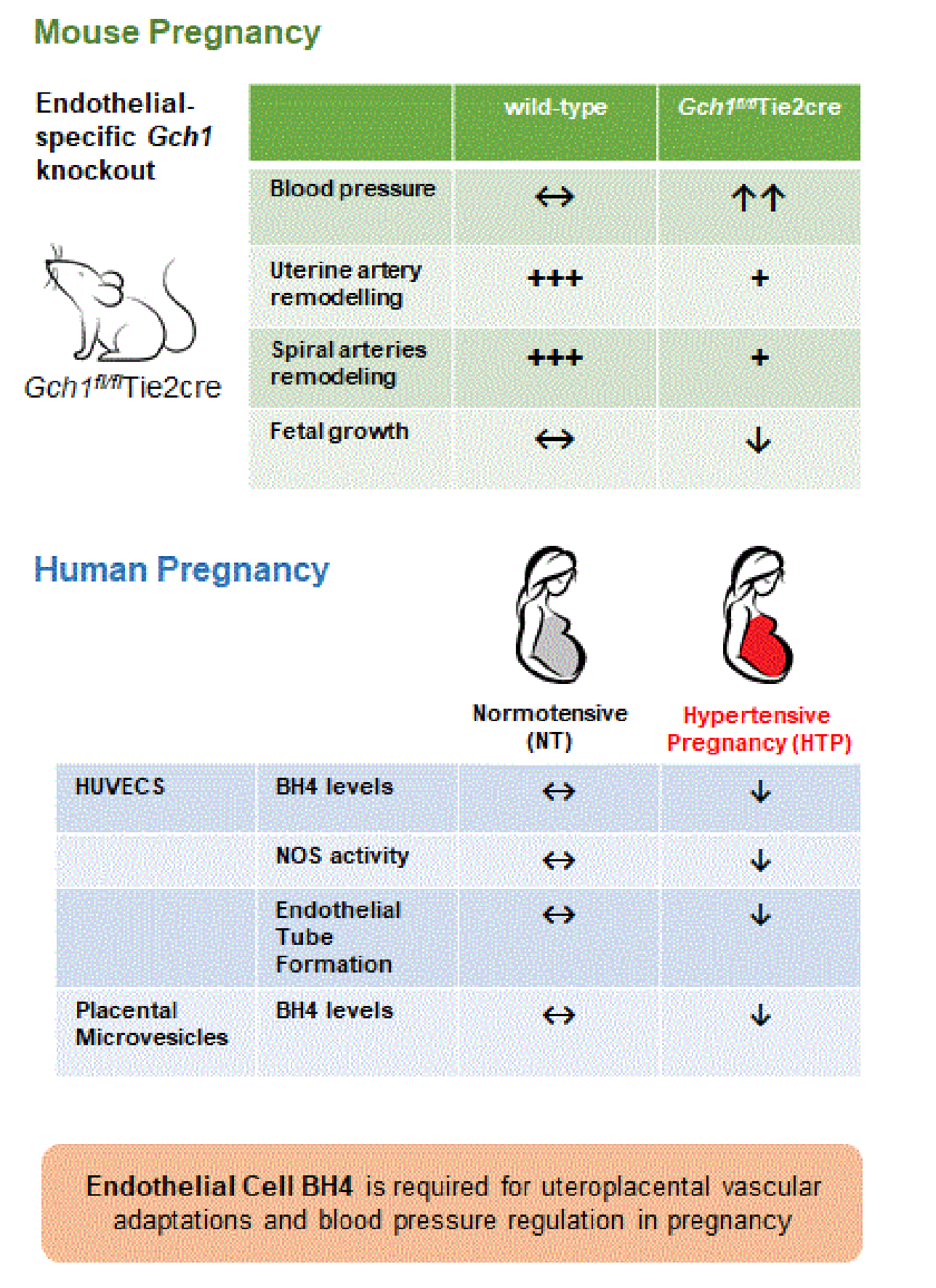

Supplement: Supplementary file 3 [file hyp-78-1871-s003.gif]
