# Supplementary material for: Endothelial GTPCH (GTP Cyclohydrolase 1) and Tetrahydrobiopterin Regulate Gestational Blood Pressure, Uteroplacental Remodeling, and Fetal Growth
Source: Hypertension. 2021 Oct 25;78(6):1871–84. doi: 10.1161/HYPERTENSIONAHA.120.17646 (PMC8577301; doi:10.1161/HYPERTENSIONAHA.120.17646)
Supplement: Supplementary file 4 [file hyp-78-1871-s004.pdf]

*** Short In Vivo Checklist**

AHA - Preclinical Animal Testing: Prevention of bias is important for experimental cardiovascular research. ***This short checklist must be completed, and the answers should be clearly presented in the manuscript as well.*** *The checklist will be used by reviewers and editors but will not be published. If a revision is requested, you will be required to complete at revision submission a more detailed checklist that will be published with the accepted article.*

This study involves animals:
Yes

Animals

Species, age, sex, strains, and sources of animals are described: Yes

Randomization

Randomization and allocation concealment were performed: Yes

Blinding

Blinding was performed: Yes

Inclusions and Exclusions (a)

Specific criteria for inclusions and exclusions are specified: Yes

Inclusions and Exclusions (b)

Criteria for inclusions and exclusions were set before the study: Yes

Reporting of Excluded Animals

All animals excluded after the randomization are reported: Yes

Statistical Methods

Statistical Methods are described: Yes

If you would like to provide a statement, please do so in this box. Otherwise, click "Generate Statement" to have one automatically generated based on the responses above. If this field is left blank, the statement will be automatically generated.

Date completed: 06/25/2021 11:09:39
User pid: 168182
